# The transcription factor GATA1 and the histone methyltransferase SET7 interact to promote VEGF-mediated angiogenesis and tumor growth and predict clinical outcome of breast cancer

**DOI:** 10.18632/oncotarget.7126

**Published:** 2016-02-02

**Authors:** Yanan Zhang, Jie Liu, Jing Lin, Lei Zhou, Yuhua Song, Bo Wei, Xiaoli Luo, Zhida Chen, Yingjie Chen, Jiaxiu Xiong, Xiaojie Xu, Lihua Ding, Qinong Ye

**Affiliations:** ^1^ Department of Medical Molecular Biology, Beijing Institute of Biotechnology, Collaborative Innovation Center for Cancer Medicine, Beijing, People's Republic of China; ^2^ Institute of Cancer Stem Cell, Cancer Center, Dalian Medical University, Liaoning, People's Republic of China; ^3^ First Affiliated Hospital, Chinese PLA General Hospital, Beijing, People's Republic of China; ^4^ Beijing Shijitan Hospital, Capital Medical University, Beijing, People's Republic of China; ^5^ The Affiliated Hospital of Qing Dao University, Qingdao, People's Republic of China; ^6^ Department of General Surgery, Chinese PLA General Hospital, Beijing, People's Republic of China

**Keywords:** VEGF, angiogenesis, tumor growth, GATA1, SET7

## Abstract

Angiogenesis is essential for tumor growth. Vascular endothelial growth factor (VEGF) is the most important regulator of tumor angiogenesis. However, how transcription factors interact with histone-modifying enzymes to regulate VEGF transcription and tumor angiogenesis remains unclear. Here, we show that transcription factor GATA1 associates with the histone methyltransferase SET7 to promote VEGF transcription and breast tumor angiogenesis. Using chromatin immunoprecipitation assay, we found that GATA1 was required for recruitment of SET7, RNA polymerase II and transcription factor II B to VEGF core promoter. GATA1 enhanced breast cancer cell (MCF7, ZR75-1 and MDA-MB-231)-secreted VEGF via SET7, which promoted vascular endothelial cell (HUVEC) proliferation, migration and tube formation. SET7 was required for GATA1-induced breast tumor angiogenesis and growth in nude mice. Immunohistochemical staining showed that expression of GATA1 and SET7 was upregulated and positively correlated with VEGF expression and microvessel number in 80 breast cancer patients. GATA1 and SET7 are independent poor prognostic factors in breast cancer. Our data provide novel insights into VEGF transcriptional regulation and suggest GATA1/SET7 as cancer therapeutic targets.

## INTRODUCTION

Angiogenesis is essential for cancer development and progression since adequate blood supply is necessary for cancer cell growth and metastasis [1, 2]. Vascular endothelial growth factor (VEGF) (also known as VEGF-A), a secreted dimeric glycoprotein, is the most important regulator of tumor angiogenesis [3]. VEGF secretion by tumor cells is a prerequisite of tumor development [4–6]. VEGF is overexpressed in a wide range of human cancers, including breast and prostate cancers, and VEGF levels inversely correlates with overall and disease-free survival in various cancers [7–9]. VEGF is thus an attractive target for cancer therapy [10].

The expression of VEGF can be regulated at the transcriptional level through the binding of transcription factors and RNA polymerase II (Pol II) to VEGF promoter [11, 12]. Transcriptional regulation of VEGF occurs via the core promoter, the minimal portion of the promoter required to properly initiate transcription, and other regulatory elements, including proximal and distal promoters. Although transcription factors, such as specific activator 1 (SP1), hypoxia-inducible factor 1 (HIF1α) and signal transducers and activators of transcription 3 (STAT3) [13–15], have been shown to regulate VEGF transcription, how the VEGF core promoter is controlled by sequence-specific transcription factors remains largely unknown. Epigenetic mechanisms also control VEGF transcription. For instance, the histone acetylase enzyme p300, the histone methyltransferase mixed lineage leukemia 1 (MLL1), and lysine specific demethylase 1 (LSD1) can regulate VEGF expression [16–20]. However, how transcription factors orchestrate the recruitment of histone modifying enzymes to VEGF promoter is unclear.

GATA1, the founding member of the GATA transcription factor family, binds to the consensus DNA sequence (T/A) GATA (A/G) with high affinity and a related sequence containing a GATC core with lower affinity [21, 22]. GATA1 plays important roles in regulation of the differentiation, proliferation, and/or apoptosis of erythroid cells and megakaryocytes. Recently, GATA1 has been shown to be overexpressed in breast carcinomas [23]. GATA1 inhibits the expression of the epithelial-mesenchymal transition marker E-cadherin and promotes breast cancer cell metastasis *in vivo* [24]. However, whether GATA1 regulates tumor angiogenesis is unclear.

In this study, we identified GATA1 as a key regulator of VEGF expression and tumor angiogenesis. GATA1 interacts with a histone methyltransferase, SET (Su(var)3–9, Enhancer of zeste, Trithorax) domain containing 7 (SETD7, SET7/9, KMT7), whose role in cancer is largely unknown [25, 26], to increase VEGF transcription by binding the VEGF core promoter and facilitating the recruitment of RNA Pol II and formation of transcription preinitiation complex. Moreover, both GATA1 and SET7 promote breast tumor growth and are independent prognostic factors of breast cancer.

## RESULTS

### Identification and characterization of GATA1 as a transcription factor regulating VEGF transcription in breast cancer cells

**Figure 1:**
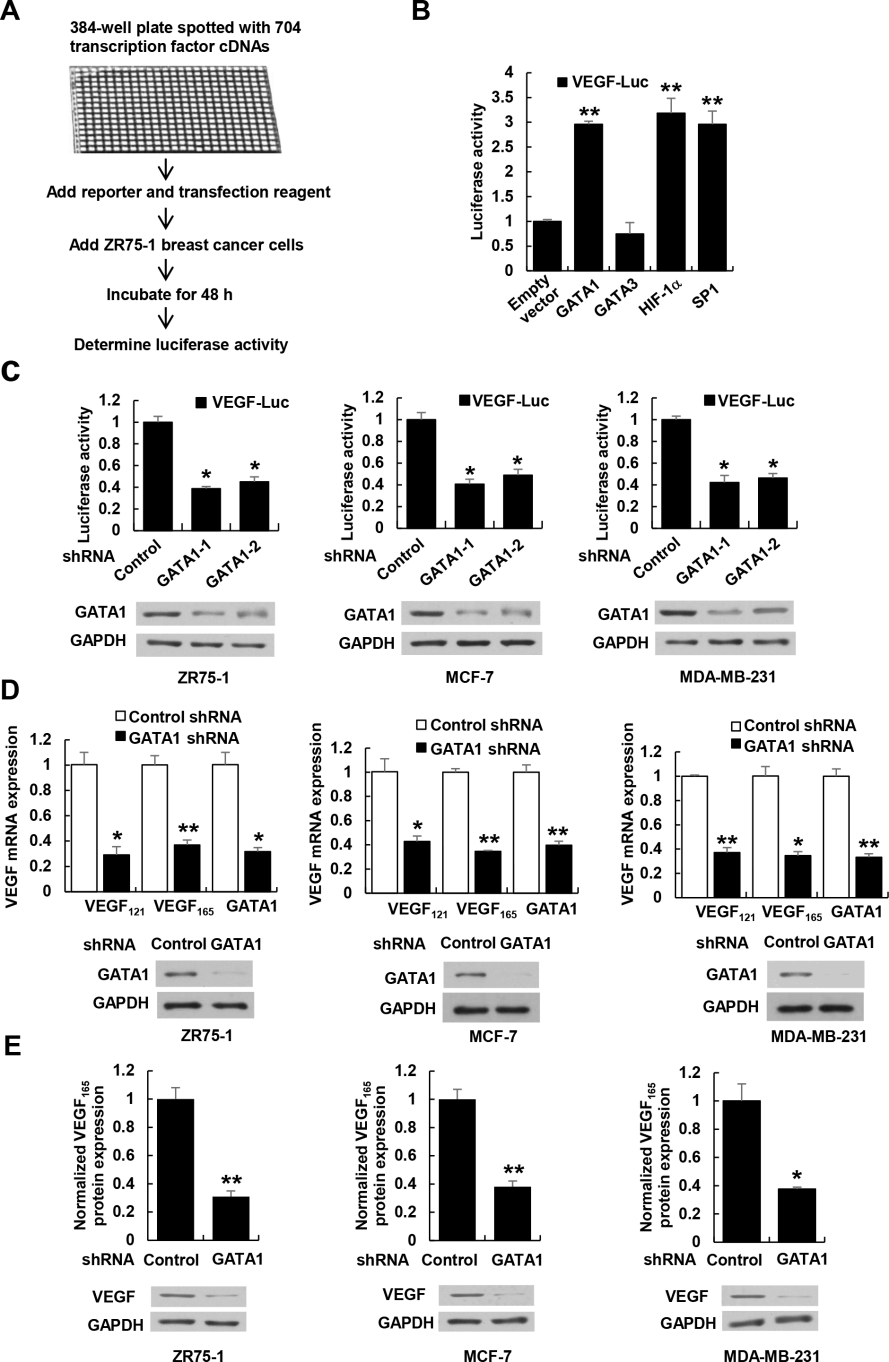
GATA1 regulates VEGF expression in breast cancer cells (**A**) Schematic illustration of screening for transcription factors that regulate VEGF-Luc reporter activity in ZR75-1 breast cancer cells. (**B**) Luciferase reporter assays in ZR75-1 breast cancer cells cotransfected with the VEGF-Luc reporter and the indicated transcription factors from (A). All values shown are expressed as the mean ± SD obtained from two independent experiments. **P < 0.01 versus empty vector. (**C**) Luciferase reporter assays in ZR75-1, MCF-7 and MDA-MB-231 breast cancer cells cotransfected with VEGF-Luc and GATA1 shRNAs or control shRNA. Representative Western blot shows the expression of GATA1. GAPDH was used as a loading control. (**D**) Real-time RT-PCR analyses of the expression of VEGF_121_ and VEGF_165_, two major VEGF isoforms, in ZR75-1, MCF-7, and MDA-MB-231 cells stably infected with lentivirus carrying GATA1 shRNA or control shRNA. Representative Western blot indicates the expression of GATA1. (**E**) VEGF concentration in cell supernatants by ELISA and VEGF protein expression by Western blot from ZR75-1, MCF7 and MDA-MB-231 cells stably infected as in (D). Data shown are mean **±** SD of triplicate measurements that have been repeated 3 times with similar results (C–E). *P < 0.05, **P < 0.01 versus control shRNA.

To identify previously unreported transcription factors regulating VEGF transcription, we used a VEGF promoter (from −2304 to +73 bp)-luciferase (VEGF-Luc) reporter to screen a transcription factor genome-wide full-length cDNA-transfection (GFC-transfection) array, consisting of 704 transfection-ready cDNA plasmids, and identified some transcription factors that stimulated the reporter gene expression in ZR75-1 breast cancer cells (Figure 1A, 1B and data not shown), such as GATA1 and the previously reported transcription factors SP1 [13] and HIF1α [14]. Although the array contained GATA3, another member of the GATA transcription factor family, it did not increase VEGF-Luc reporter activity (Figure 1B and Supplementary Figure S1A), indicating that GATA1 specifically stimulate VEGF-Luc reporter activity. We further confirmed GATA1 overexpression-mediated enhancement of VEGF-Luc reporter activity using our GATA1 expression construct in ZR75-1, MCF-7 and MDA-MB-231 breast cancer cells (Supplementary Figure S1A). In contrast, GATA1 knockdown decreased VEGF-Luc reporter activity in these cells (Figure 1C). GATA1 overexpression increased VEGF-Luc reporter activity independent of oxygen although hypoxia increased the reporter activity (Supplementary Figure S1B). Consistent with the results of the luciferase reporter analysis, GATA1 overexpression increased VEGF mRNA expression (Supplementary Figure S1C) and VEGF secretion level (Supplementary Figure S1D), whereas GATA1 knockdown reduced the level of VEGF mRNA (Figure 1D) and secretion of endogenous VEGF protein (Figure 1E).

### Cancer cell-secreted VEGF regulated by GATA1 controls human umbilical vascular endothelial cell (HUVEC) proliferation and migration

**Figure 2:**
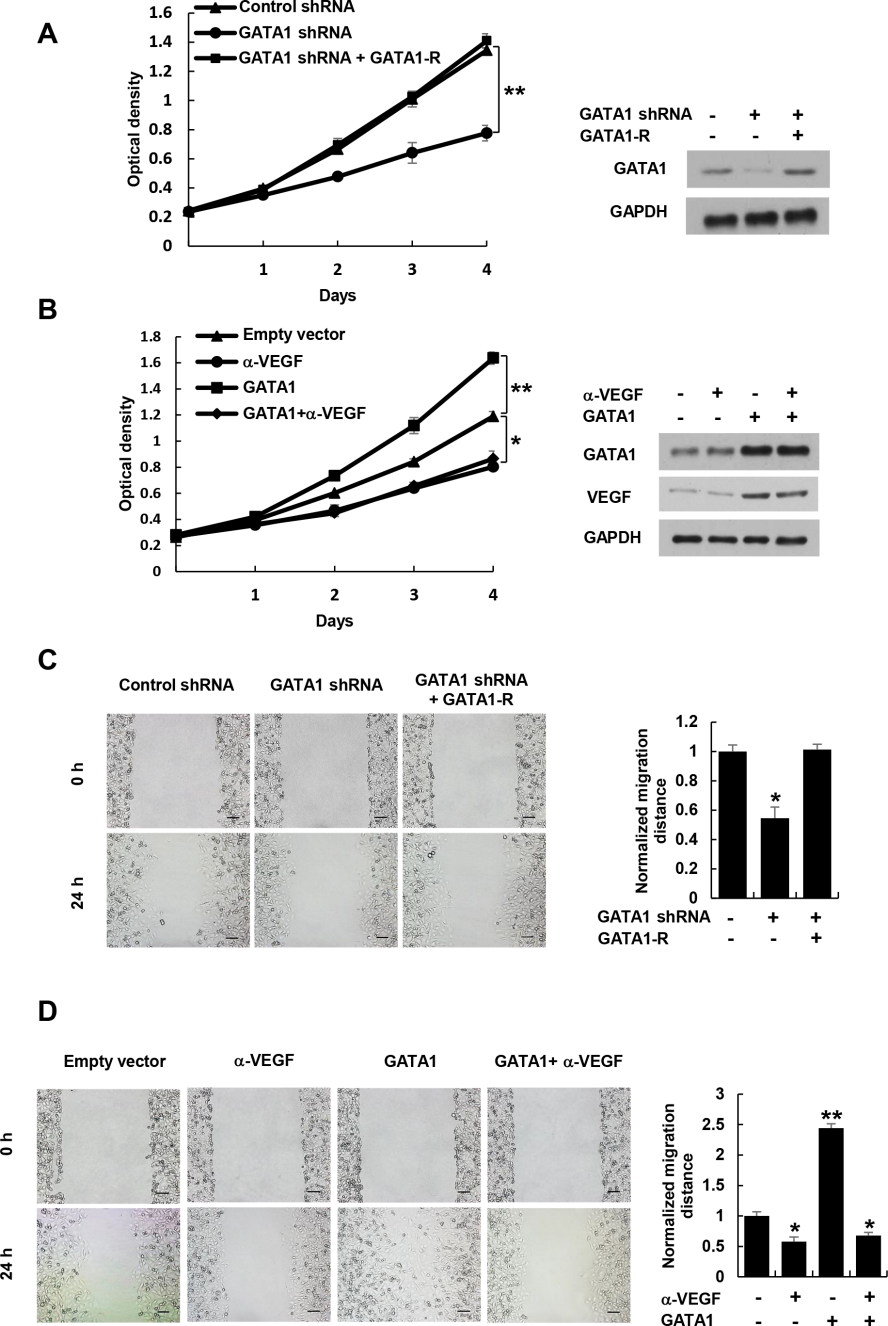
Cancer cell-secreted VEGF regulated by GATA1 modulates HUVEC proliferation and migration (**A**) Cell proliferation assays in HUVEC cells cultured in conditioned medium from MCF7 cells stably infected with lentivirus carrying GATA1 shRNA or GATA1 shRNA plus shRNA-resistant GATA1 (GATA1-R). Representative Western blot shows the expression of GATA1. (**B**) Cell proliferation assays in HUVEC cells cultured in conditioned medium from MCF7 cells stably transfected with GATA1 and treated with a VEGF neutralizing antibody (α-VEGF). Representative Western blot indicates the expression of GATA1 and VEGF. *P < 0.05, **P < 0.01 (A, B). (**C**) Wound healing assays for HUVEC cells cultured in conditioned medium from MCF7 cells stably infected as in (A). (**D**) Wound healing assays for HUVEC cells cultured in conditioned medium from MCF7 cells stably transfected and treated as in (B). The image displayed is one of the representative results (C, D). Scale bar: 100 μm. All values shown are mean ± SD of triplicate measurements and have been repeated 3 times with similar results (C, D). *P < 0.05, **P < 0.01 versus control shRNA or empty vector.

Most types of cells, including cancer cells, but usually not endothelial cells themselves, secrete VEGF. Secreted VEGF plays critical roles in regulation of endothelial cell proliferation and migration [4–6]. Since GATA1 promotes VEGF secretion in breast cancer cells, we determined the effect of the conditioned medium derived from GATA1 overexpression or knockdown stable breast cancer cell lines on HUVEC proliferation and migration. The conditioned medium from GATA1 knockdown MCF7, ZR75-1 and MDA-MB-231 cells decreased HUVEC proliferation compared with control medium (Figure 2A and Supplementary Figure S2A, S2B). These effects could be rescued by the conditioned medium from these cells re-expressing GATA1 (Figure 2A and Supplementary Figure S2A, S2B). Neutralization of secreted VEGF by a VEGF neutralizing antibody abolished the ability of the conditioned medium from GATA1-overexpressing breast cancer cells to increase HUVEC proliferation (Figure 2B and Supplementary Figure S2C, S2D), suggesting that GATA1-mediated enhancement of VEGF expression in the conditioned medium is responsible for HUVEC proliferation. Similar trends were observed in HUVEC migration experiments (Figure 2C, 2D and Supplementary Figure S3). Increased HUVEC migration regulated by GATA1-mediated enhancement of VEGF expression is not due to increased HUVEC proliferation because there was no significant difference in HUVEC proliferation within 24 h of culture between the conditioned media from GATA1-overexpressing cancer cells and control cells (Figure 2B and Supplementary Figure S2C, S2D).

### Cancer cell-secreted VEGF regulated by GATA1 controls HUVEC tube formation and angiogenesis

**Figure 3:**
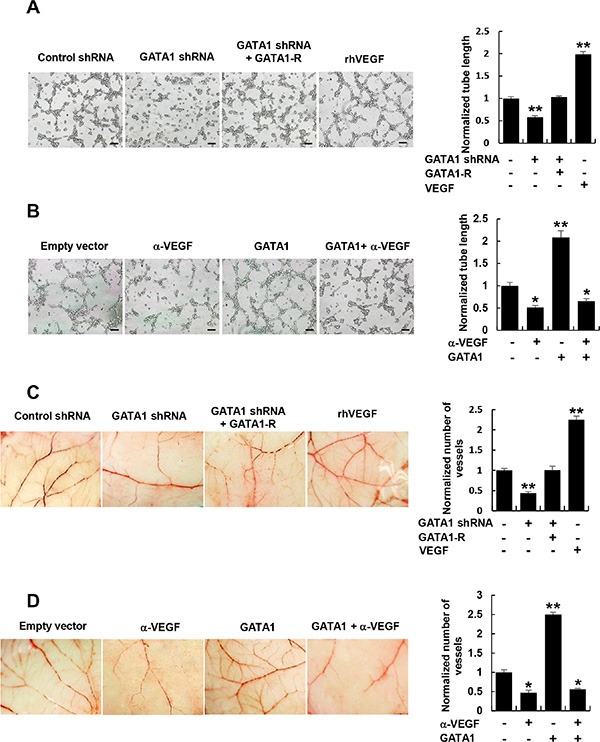
Cancer cell-secreted VEGF regulated by GATA1 modulates HUVEC tube formation and angiogenesis (**A**) Tube formation assays for HUVEC cells cultured in the conditioned medium from MCF7 cells stably infected as in Figure 2A. (**B**) Tube formation assays for HUVEC cells cultured in the conditioned medium from MCF7 cells stably transfected and treated as in Figure 2B. (**C**) CAM assays with conditioned medium from MCF7 cells stably infected as in Figure 2A. (**D**) CAM assays with conditioned medium from MCF7 cells stably transfected and treated as in Figure 2B. The image displayed is one of the representative results (A–D). Scale bar: 100 μm (A, B). Recombinant human (rh) VEGF (10 ng/ml) (PeproTech) was used as a positive control (A and C). All values shown are mean ± SD of triplicate measurements and have been repeated 3 times with similar results (A–D). *P < 0.05, **P < 0.01 versus control shRNA or empty vector.

The key aspect of angiogenesis is the formation of capillary tubes by endothelial cells [27]. Thus, we first tested if GATA1-mediated VEGF expression affects HUVEC tube formation *in vitro*. The conditioned medium from GATA1 knockdown breast cancer cells inhibited HUVEC tube formation (Figure 3A and Supplementary Figure S4A, S4B). The effects could be rescued by GATA1 reexpression in the GATA1 knockdown cells. Inhibition of cancer cell-secreted VEGF by a VEGF neutralizing antibody abrogated the ability of the conditioned medium from GATA1-overexpressing breast cancer cells to increase HUVEC tube formation (Figure 3B and Supplementary Figure S4C, S4D).

Next, we determined the effect of GATA1-mediated VEGF expression on *in vivo* angiogenesis by chick embryo chorioallantoic membrane (CAM) assay. CAM treated with the conditioned medium from GATA1 knockdown breast cancer cells had reduced number of new blood vessels and reexpression of GATA1 in GATA1 knockdown cells abolished this effect (Figure 3C and Supplementary Figure S5A, S5B). Importantly, Neutralization of cancer cell-secreted VEGF by a VEGF neutralizing antibody abolished the ability of the conditioned medium from GATA1-overexpressing breast cancer cells to promote the formation of new blood vessels on the CAM (Figure 3D and Supplementary Figure S5C, S5D). As a positive control, recombinant human VEGF promoted HUVEC tube formation and angiogenesis on the CAM. Taken together, these data indicate that GATA1-mediated enhancement of VEGF expression in the conditioned medium is required for HUVEC tube formation and angiogenesis.

### GATA1 recruits the histone methyltransferase SET7 and RNA polymerase II to the VEGF core promoter

**Figure 4:**
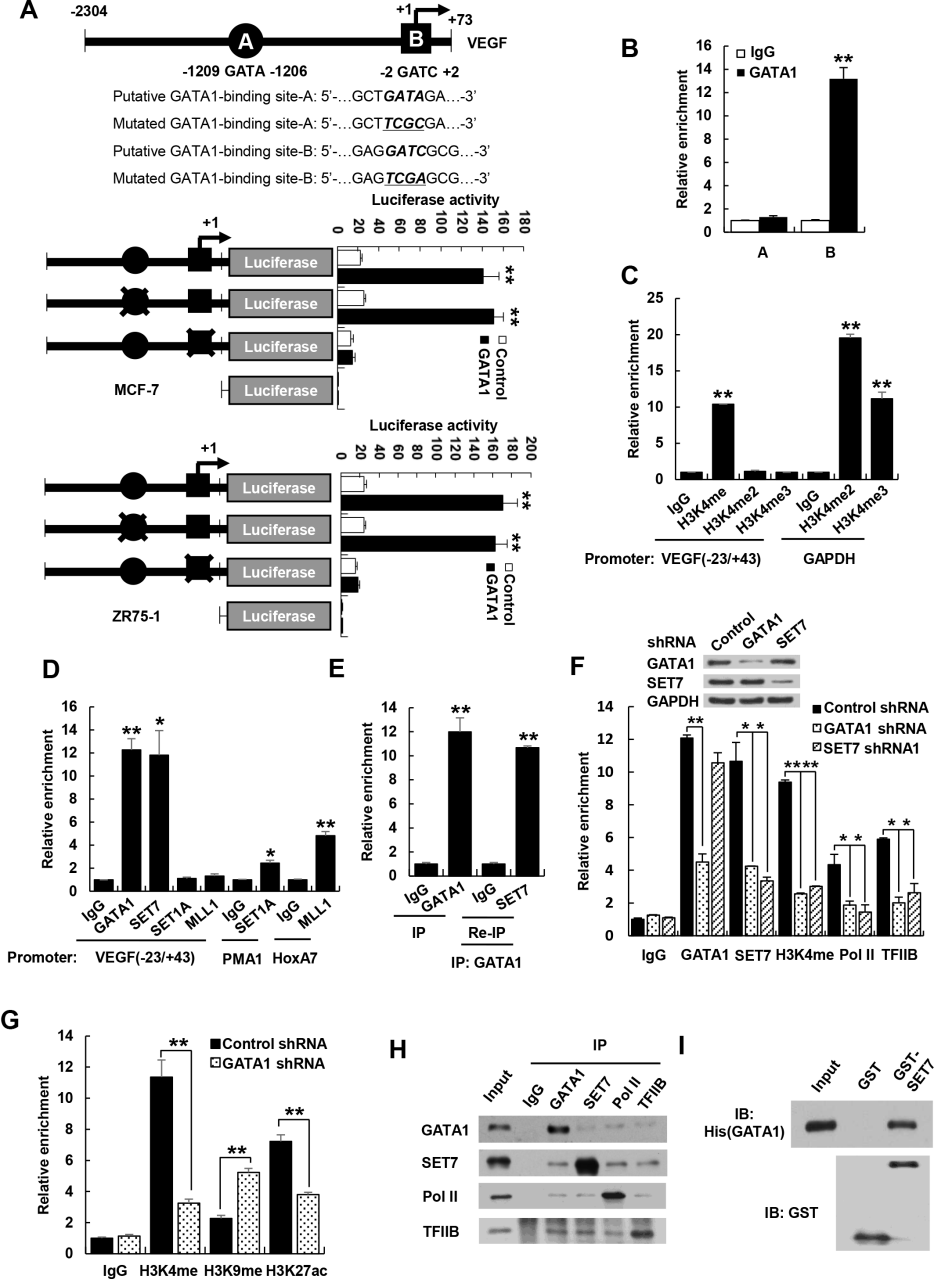
GATA1 is required for recruitment of SET7 and RNA polymerase II to VEGF promoter (**A**) Luciferase activity of different VEGF promoter constructs in MCF7 and ZR75-1 cells transfected with GATA1 or empty vector. The arrow indicates the position of the transcriptional start site. A and B are putative GATA1 binding sites. the “X” shows the mutated GATA1-binding site. Data shown are mean ± SD of triplicate measurements and have been repeated 3 times with similar results. **P < 0.01 versus empty vector with corresponding promoter reporter. (**B**) ChIP analysis of the occupancy of GATA1 on the putative GATA1 binding sites of VEGF promoter in MCF7 cells. (**C**) ChIP analysis of the occupancy of H3K4me, H3K4me2 and H3K4me3 on VEGF promoter containing GATC site (−23/+43) in MCF7 cells. GAPDH promoter was used as positive controls for H3K4me2 and H3K4me3. (**D**) ChIP analysis of the occupancy of GATA1 and different histone methyltransferases on VEGF promoter (−23/+43) in MCF7 cells. PMA1 and HoxA7 promoters were used as positive controls for SET1 and MLL1, respectively. (**E**) Re-ChIP analysis of the occupancy of GATA1 and SET7 on VEGF promoter (−23/+43) in MCF7 cells. (**F**) ChIP analysis of MCF7 cells stably infected with lentivirus carrying GATA1 shRNA or SET7 shRNA1 on VEGF promoter (−23/+43) with the indicated antibodies. Western blot shows the knockdown effects of GATA1 and SET7. All values shown are mean ± SD of triplicate measurements and have been repeated 3 times with similar results (B–F). *P < 0.05, **P < 0.01 versus corresponding control (B–F). (**G**) ChIP analysis of the occupancy of H3K4me, H3K9me and H3K27ac on VEGF promoter containing GATC site (−23/+43) in MCF7 cells. **P < 0.01 versus corresponding control. (**H**) Reciprocal coimmunoprecipitation analysis of endogenous interactions among GATA1, SET7, Pol II and TFIIB. (**I**) GST pull-down analysis of direct interaction between GATA1 and SET7. Purified His-tagged GATA1 and GST-SET7 or GST were used.

To investigate how GATA1 promotes VEGF transcription in breast cancer cells, we first determined the binding site of GATA1 on the VEGF promoter. GATA1 has been shown to bind GATA or GATC motif [21, 22]. Indeed, nucleotides from −1209 to −1206 bp (GATA) and −2 to +2 bp (GATC) on the VEGF promoter contains putative binding site of GATA1 (Figure 4A). We used promoter reporter mutation analysis to identify binding site of GATA1 in the VEGF promoter. The GATC site, but not the GATA site, was responsible for GATA1 modulation of VEGF promoter reporter activity, because GATA1 increased the activity of the reporter with the GATC site or with the mutated GATA site, but not with the mutated GATC site in MCF-7 and ZR75-1 cells (Figure 4A). Chromatin immunoprecipitation (ChIP) assay indicated that GATA1 was recruited to the region containing the GATC site, but not the GATA site, of the VEGF promoter (Figure 4B). Moreover, electrophoretic mobility shift assay (EMSA) showed that GATA1 bound to the GATC site, but not the mutant GATC site or the GATA site, *in vitro* (Supplementary Figure S6A). The binding was specifically inhibited by a 100-fold molar excess of an unlabeled probe corresponding to the GATC site. These data indicate that GATA1 binds to the GATC site of the VEGF promoter. To pinpoint which domain of GATA1 is involved in binding of the GATC motif, GATA1 deletion mutants were tested in the EMSA assay. GATA1 (252−413) containing C-terminal zinc finger (CF) mediated binding to the GATC motif, while GATA1 (1−150) containing activation domain and GATA1 (151−251) containing N-terminal zinc finger (NF) did not (Supplementary Figure S6B).

Histone methylation can lead to transcriptional repression or activation, depending on the target sites [28, 29]. Methylation of histone H3 on lysine 4 (H3K4) generally correlates with transcriptional activation. Since GATA1 promotes VEGF transcription, we examined whether H3K4 methylation is enriched at the region containing the GATC site. H3K4 monomethylation (H3K4me), but not H3K4 dimethylation (H3K4me2) and trimethylation (H3K4me3), was enriched at the GATC site, although H3K4me2 and H3K4me3 were enriched at GAPDH promoters, which were used as positive controls in the ChIP kits (Figure 4C).

Next, we investigated which histone methyltransferase is responsible for the monomethylation of H3K4 on the region containing GATC site (−23/+43 bp). Like GATA1, SET7, which only monomethylates H3K4 [30], was recruited to the GATC site of VEGF promoter (Figure 4D). Mixed lineage leukemia protein 1 (MLL1) and SET1A, both of which can catalyze mono-, di-, and trimethylation of H3K4 [25], were not recruited to the GATC site, although MLL1 and SET1A were recruited to the previously reported promoters homeobox-containing 7 (HoxA7) [31] and plasma membrane ATPase 1 (PMA1) [32], respectively (Figure 4D). SET7 did not methylate GATA1 although it methylated the previous reported transcription factor estrogen receptor α (Supplementary Figure S6C). Re-ChIP experiments showed that GATA1 associated with SET7 on the GATC site of VEGF promoter (Figure 4E and Supplementary Figure S6D). Importantly, Consistent with the results of GATA1 activation of VEGF transcription, GATA1 knockdown reduced recruitment of SET7, H3K4me, RNA polymerase II (Pol II), and TFIIB, a general transcription factor that is involved in the formation of the Pol II preinitiation complex and aids in stimulating transcription initiation [33, 34], to the GATC site (Figure 4F and Supplementary Figure S6E). SET7 knockdown caused a marked reduction of recruitment of H3K4me and RNA polymerase II to the GATC site of VEGF promoter but had no effect on recruitment of GATA1 to the GATC site (Figure 4F and Supplementary Figure S6E). In addition, knockdown of GATA1 or SET7 had no effects on recruitment of RNA polymerase II and H3K4me to the VEGF promoter regions −1241∼-1174, −206∼−114, −113∼−6, +49∼+109, +132∼+244, and +295∼+349 (Supplementary Figure S6F and S6G). Besides that GATA1 knockdown reduces recruitment of H3K4me on VEGF promoter, knockdown of GATA1 increased H3K9me, a repressive mark of transcription, and decreased acetylation of H3K27 (H3K27ac), an active mark of transcription (Figure 4G).

Based on our findings that GATA1 regulates the recruitment of SET7, Pol II, and TFIIB, we first tested whether GATA1 physically interacts with SET7. Indeed, endogenous GATA1 specifically coimmunoprecipitated with endogenous SET7 in MCF7 cells (Figure 4H). The interaction between GATA1 and SET7 is direct because purified His-tagged GATA1 protein interacted with purified GST-SET7 but not GST alone (Figure 4I). GATA1 (252–413) containing the CF, but not other GATA1 deletion mutants, associated with SET7 (Supplementary Figure S7A). SET7 (1–107) containing N-terminal fragment (NF) interacted with GATA1, whereas SET7 (108–214) containing middle region fragment (MF) and SET7 (215–366) containing SET domain did not (Supplementary Figure S7B). Unexpectedly, the molecular weight of SET7 (1–107) was larger than that predicted by sequence data. This might be caused by posttranslational modifications because its coding sequence and open reading frame was correct according to the results of DNA sequencing (data not shown). Moreover, reciprocal coimmunoprecipitation experiments showed that GATA1, SET7, Pol II, and TFIIB interacted with each other (Figure 4H). GATA1 did not interact with HIF1α although HIF1α interacted with four and a half LIM protein 1 (FHL1) as we previously reported [49] (Supplementary Figure S7C). Taken together, these results suggest that GATA1, SET7, Pol II, and TFIIB may form a preinitiation complex on the GATC site of VEGF promoter.

### GATA1 regulates VEGF expression and VEGF-induced HUVEC proliferation, migration and tube formation as well as angiogenesis via SET7

**Figure 5:**
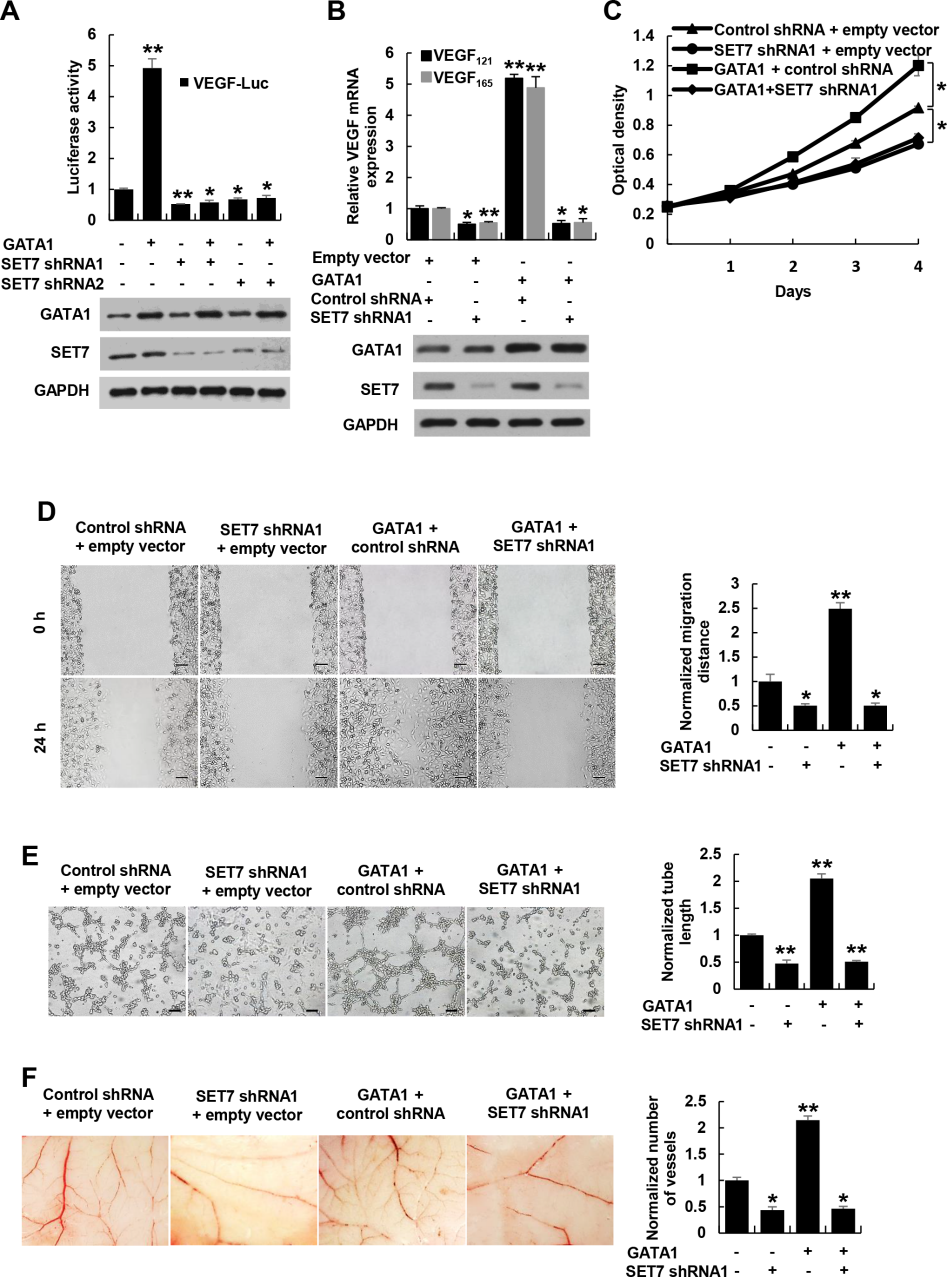
GATA1 modulates VEGF expression and VEGF-induced HUVEC proliferation, migration and tube formation as well as angiogenesis through SET7 (**A**) Luciferase reporter assays in MCF7 cells cotransfected with the VEGF-Luc reporter, GATA1 and SET7 shRNA1, SET7 shRNA2 or control shRNA. Representative Western blot shows the expression of GATA1 and SET7. (**B**) Real-time RT-PCR analysis of the VEGF_121_ and VEGF_165_ expression in MCF7 cells stably infected with lentiviruses carrying GATA1 and SET7 shRNA. Representative Western blot indicates the expression of GATA1 and SET7. (**C**) Cell proliferation assays in HUVEC cells cultured in conditioned medium from MCF7 cells stably infected as in (B). (**D**) Wound healing assays for HUVEC cells cultured in conditioned medium from MCF7 cells stably infected as in (B). Scale bar: 100 μm. (**E**) Tube formation assays for HUVEC cells cultured in conditioned medium from MCF7 cells stably infected as in (B). Scale bar: 100 μm. (**F**) CAM assays with conditioned medium from MCF7 cells stably infected as in (B). All values shown are mean ± SD of triplicate measurements and have been repeated 3 times with similar results (A–F). *P < 0.05, **P < 0.01 versus corresponding control.

Since SET7 is critical for recruitment of H3K4me, Pol II and TFIIB to GATA1 binding site, we tested whether GATA1 regulates VEGF transcription via SET7. Knockdown of SET7 decreased VEGF-Luc reporter activity (Figure 5A) and VEGF mRNA expression (Figure 5B and Supplementary Figure S8A) in breast cancer cells. Importantly, SET7 knockdown abolished the ability of GATA1 to increase VEGF-Luc reporter activity and VEGF mRNA expression. Moreover, SET7 and GATA1 synergistically increased VEGF-Luc reporter activity (Supplementary Figure S8B) and VEGF mRNA expression (Supplementary Figure S8C), whereas the methyltransferase-deficient mutant Set7 (H297G) failed to promote GATA1-induced VEGF-Luc reporter activity and VEGF mRNA expression, suggesting that the enzymatic activity of SET7 is required for GATA1 modulation of VEGF transcription.

Knockdown of the VEGF receptor VEGFR2 did not affect the ability of GATA1 and SET7 to promote VEGF expression (Supplementary Figure S10C). On the other hand, GATA1 knockdown attenuated but not abrogated SET7-mediated enhancement of VEGF mRNA expression, suggesting that other transcription factors may also interact with SET7 to promote VEGF expression (Supplementary Figure S8D).

Next, we determined whether SET7 plays a role in GATA1 modulation of VEGF-induced HUVEC proliferation, migration and tube formation as well as angiogenesis. The conditioned medium from GATA1-overexpreesing breast cancer cells increased HUVEC proliferation, migration and tube formation as well as angiogenesis in the CAM model (Figure 5C–5F and Supplementary Figure S9A–S9D). Opposite results were observed with the conditioned medium from SET7 knockdown breast cancer cells. Importantly, the conditioned medium from GATA1-overexpreesing breast cancer cells with SET7 knockdown abolished the ability of the conditioned medium from GATA1-overexpreesing breast cancer cells to stimulate HUVEC proliferation, migration and tube formation as well as angiogenesis (Figure 5C–5F and Supplementary Figure S9A–S9D), suggesting that GATA1 promotes VEGF-induced HUVEC proliferation, migration and tube formation as well as angiogenesis via SET7. Moreover, VEGFR2 knockdown in HUVEC cells abolished the ability of the conditioned medium from GATA1- or SET7-overexpreesing breast cancer cells to stimulate vascular tube formation (Supplementary Figure S10D). However, the conditioned medium from GATA1- or SET7-overexpreesing breast cancer cells with VEGFR2 knockdown had similar effect on angiogenesis to the conditioned medium from GATA1- or SET7-overexpreesing breast cancer cells (Supplementary Figure S10E), suggesting that VEGFR2 is not required for GATA1 or SET7 modulation of VEGF expression in breast cancer cells.

### GATA1 modulates breast cancer cell growth through SET7

**Figure 6:**
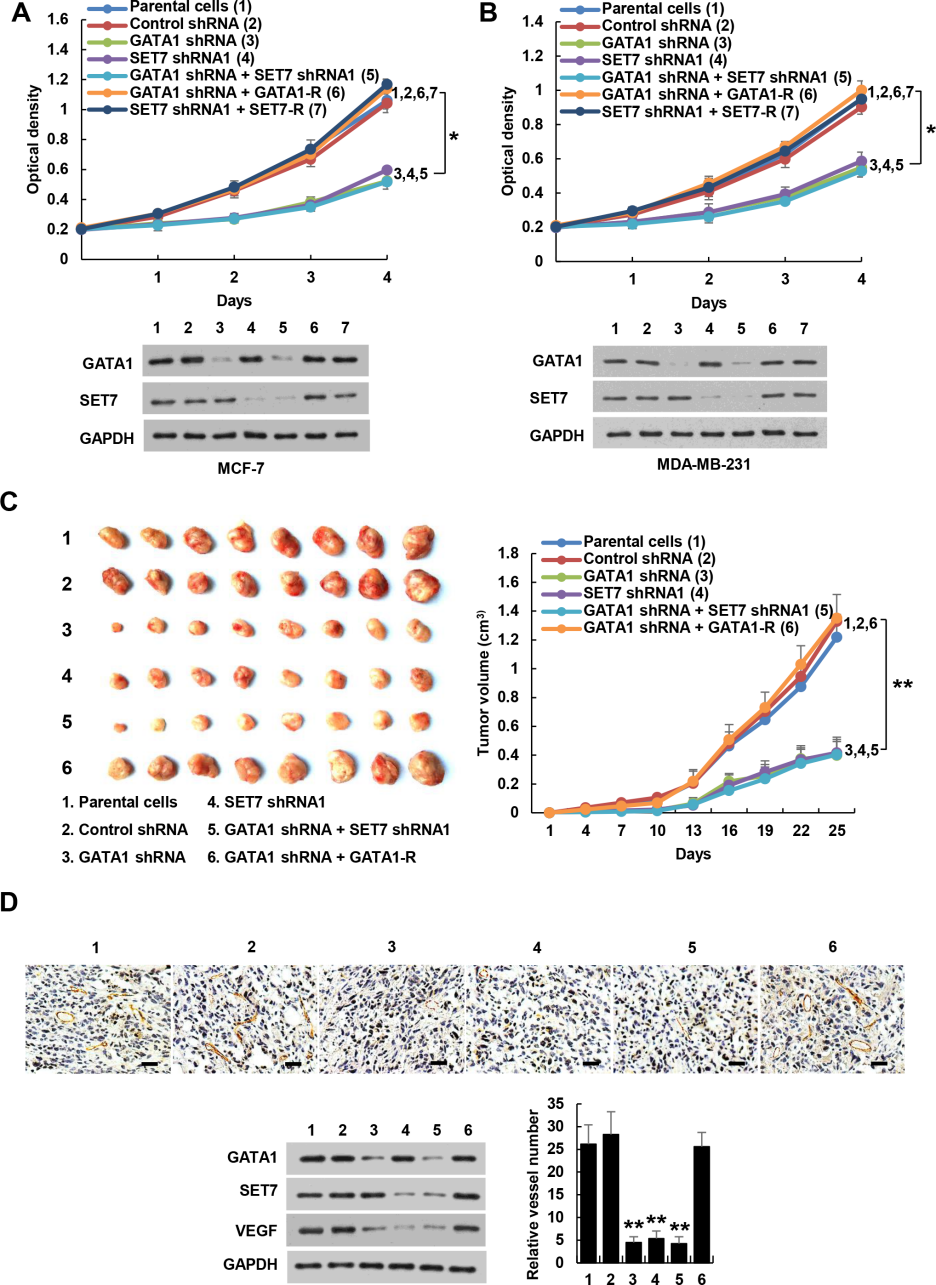
GATA1 regulates breast cancer cell proliferation through SET7 (**A**), (**B**) Cell proliferation assays in MCF7 (A) and MDA-MB-231 cells (B) stably infected with lentiviruses carrying the indicated constructs. Western blot shows the expression of GATA1 and SET7. All values shown are mean ± SD of triplicate measurements and have been repeated 3 times with similar results. *P < 0.05 on day 4. (**C**) Volume of xenograft tumors derived from MDA-MB-231 cells stably infected as in (A). Data are shown as mean ± SD (*n* = 8). **P < 0.01 on day 25. (**D**) Representative tumor tissues were subjected to immunohistochemical staining with an anti-CD31 antibody and immunoblot with the indicated antibodies. Pictures from eight areas in each group were taken and the vessel number was manually counted. Scale bar: 50 μm.

Breast cancer cells have been shown to produce VEGF not only for proliferation of endothelial cells but also for proliferation and/or survival of cancer cells in an autocrine and/or paracrine manner [35, 36]. Solid tumors cannot grow larger than 2 mm in diameter without angiogenesis. Thus, we tested the effects of GATA1 and SET7 on cancer cell proliferation in culture and tumor growth in a xenograft mouse model. Knockdown of GATA1 or SET7 inhibited proliferation of MCF7 and MDA-MB-231 cells (Figure 6A, 6B). These effects could be rescued by GATA1 or SET7 reexpression in the GATA1 or SET7 knockdown cells. SET7 knockdown abolished the ability of GATA1 knockdown to inhibit proliferation of MCF7 and MDA-MB-231 cells (Figure 6A, 6B). SET7 knockdown also abrogated the ability of GATA1 overexpression to enhance proliferation of MCF7 and MDA-MB-231 cells, suggesting that GATA1-mediated breast cancer cell proliferation is dependent on SET7 (Supplementary Figure S10A, S10B). Moreover, VEGFR2 knockdown abrogated the ability of GATA1 and SET7 to promote VEGF-mediated cancer cell growth (Supplementary Figure S10C).

GATA1 was shown to be essential for erythroid cell survival by activating the expression of the oncogene bcl-xL [37]. However, GATA1 inhibits Myb oncogene expression when red cell progenitors differentiate into erythrocytes [38], and it also represses Myc oncogene expression during erythroid maturation [39]. In MCF7 and MDA-MB-231 breast cancer cells, overexpression of GATA1 or SET7 increased the transcription of VEGF, bcl-xL, and Myc, whereas knockdown of GATA1 or SET7 decreased that of VEGF, bcl-xL, and Myc (Supplementary Figure S11). GATA1 had no effect on Myb transcription. These data suggest that GATA1 exerts tissue-specific transcriptional activity.

Consistent with the results of the cell proliferation assays, mice inoculated with GATA1 or SET7 knockdown MDA-MB-231 cells grew more slowly than those cells stably infected with control shRNA and parental cells (Figure 6C). Again, the inhibitory effect of GATA1 knockdown on tumor growth could be rescued by GATA1 reexpression in the GATA1 knockdown cells. SET7 knockdown abrogated the ability of GATA1 knockdown to repress MDA-MB-231 tumor growth (Figure 6C). Immunohistochemical staining with an antibody against CD31, a vascular marker, and Western blot with VEGF antibody showed that knockdown of GATA1 or SET7 inhibited VEGF expression and angiogenesis, and SET7 knockdown abolished the effects of GATA1 on VEGF expression and angiogenesis (Figure 6D). Taken together, these data indicate that GATA1 regulates breast cancer cell growth and tumor angiogenesis through SET7.

### GATA1 and SET7 positively correlate with VEGF expression and are independent prognostic markers for breast cancer

**Figure 7:**
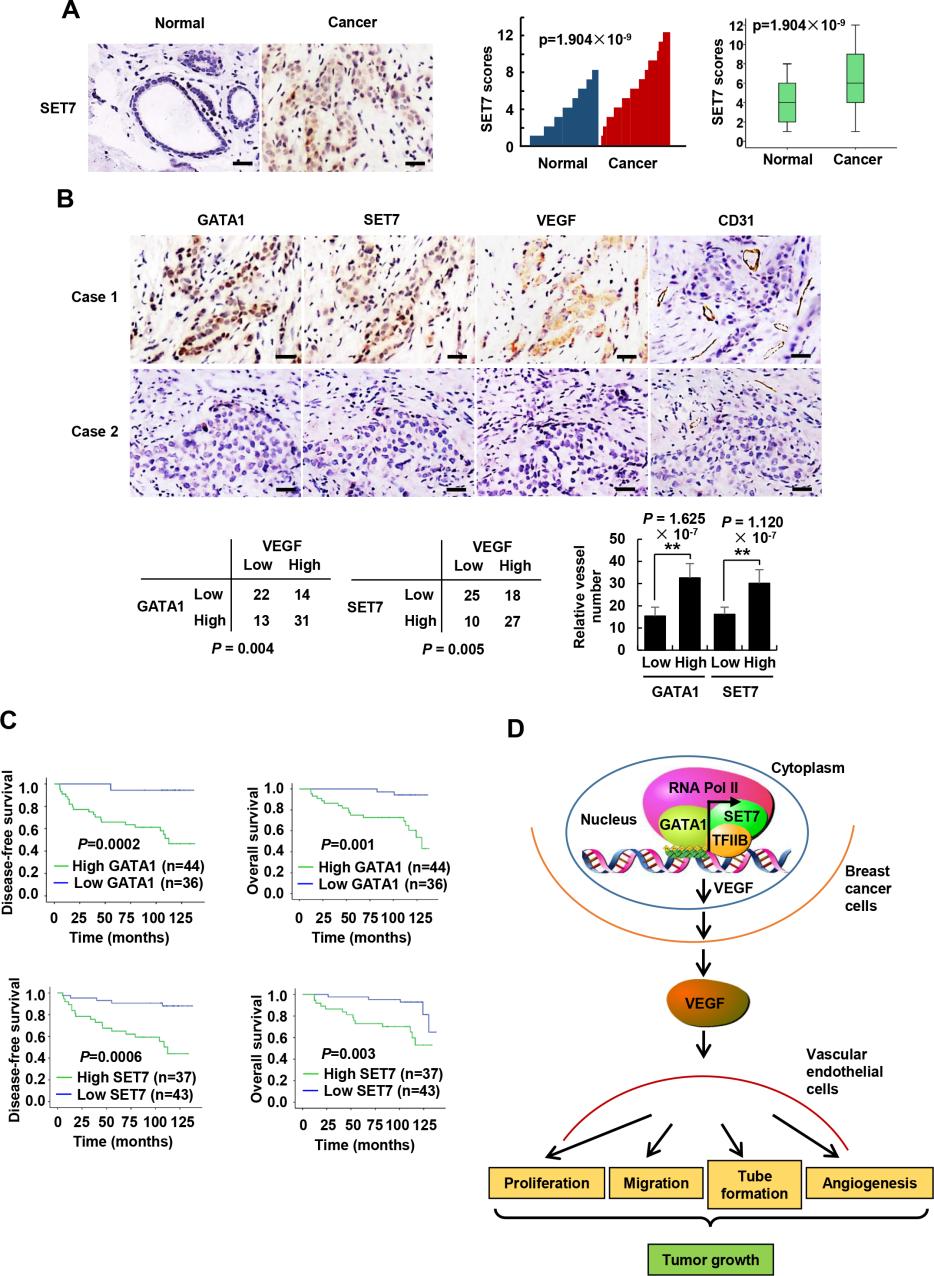
GATA1 and SET7 positively correlate with VEGF expression and are independent poor prognostic markers for breast cancer (**A**) Representative immunohistochemical staining of SET7 in human cancerous breast tissues and adjacent normal breast tissues. Scale bar: 25 μm. SET7 expression scores were plotted and compared (Mann-Whitney *U* test). (**B**) Representative immunohistochemical staining of GATA1, SET7, VEGF and CD31 in human breast cancer samples. Scale bar: 25 μm. For the quantification of microvessel density, pictures from eight areas in each tissue were taken and the vessel number was counted. The correlation of GATA1 or SET7 with VEGF or microvessel number (positive CD31 staining) is shown. The *P* value was generated using Pearson's *χ2* test (GATA1, SET7 and VEGF) and Wilcoxon ranked sum test (CD31). (**C**) Kaplan-Meier estimates of disease-free survival (left panel) and overall survival (right panel) of breast cancer patients. Marks on graph lines represent censored samples. (**D**) Proposed model for GATA1 modulation of VEGF expression as well as its tumor-promoting function. GATA1 recruits SET7, TFIIB and RNA Pol II to VEGF core promoter, resulting in increased VEGF transcription and secretion in breast cancer cells. The cancer cell-secreted VEGF enhances vascular endothelial cell proliferation, migration and tube formation as well as tumor angiogenesis and growth.

To explore the clinical significance of GATA1 and SET7, we conducted immunohistochemistry (IHC) of 80 human breast tumor samples. We confirmed the specificity of the antibodies for GATA1 and SET7 used in IHC by antigen competition and immunoblotting of lysates from MCF7 and ZR75-1 breast cancer cells transfected with GATA1 and SET7 siRNA (Supplementary Figure S12). Consistent with previous report [23], GATA1 was overexpressed in breast cancer patients (data not shown). Intriguingly, compared with their corresponding nontumorous counterparts, SET7 expression was also upregulated in breast cancer tissues (*P* = 1.904 × 10^−9^) (Figure 7A). We next examined associations between GATA1/SET7 expression and VEGF expression or microvessel number. Expression of both GATA1 and SET7 positively correlated with VEGF expression in 80 human breast cancer tissues (*P* = 0.004 and *P* = 0.005, respectively). However, in 1100 cases of breast cancer patients from TCGA breast cancer patient data set, we did not find correlation between expression of GATA1 mRNA or SET7 mRNA with VEGF mRNA expression. Such difference may be due to the fact that mRNA levels do not always correlate with protein levels. Our IHC results further showed that tumors with high GATA1 and SET7 expression had significantly greater microvessel number than those with low GATA1 and SET7 expression (*P* = 1.625 × 10^−7^ for GATA1 and *P* = 1.120 × 10^−7^ for SET7) (Figure 7B). GATA1/SET7 expression positively associated with tumor size, nodal status and grade, and inversely correlated with the expression of estrogen receptor α (ERα), but they did not associate with age, progesterone receptor (PR) and human epidermal growth factor receptor 2 (HER2) (Supplementary Table S2). Moreover, we observed a strong correlation of higher GATA1 and SET7 expression with shorter disease-free (*P* = 0.0002 and *P* = 0.0006, respectively) and overall survival (*P* = 0.001 and *P* = 0.003, respectively) (Figure 7C). After univariate analysis by Cox proportional hazards model (Supplementary Table S1), tumor size, nodal status, grade, and ER, GATA1, and SET7 status were demonstrated as significant prognostic parameters for disease-free survival (DFS) and overall survival (OS). PR was a significant prognostic factor of DFS. A multivariate analysis revealed that tumor size, nodal status, grade, and GATA1 and SET7 status were independent poor prognostic factors of DFS and OS. ER**α** was an independent favorable prognostic factor of OS (Supplementary Table S1). Taken together, these findings implicated the significance of GATA1 and SET7 in breast cancer prognosis.

## DISCUSSION

Transcription factors can orchestrate the recruitment of histone modifying complexes to specific sets of target genes [40–42]. However, the identity of relevant histone modifying enzymes that each transcription factor interacts with is still largely unexplored. Our study documents a functional role for transcription factor GATA1 in recruiting the histone methyltransferase SET7 to specific gene targets, such as VEGF. SET7 acts as a coactivator for GATA1 in regulation of VEGF transcription. Although SET7 can methylate transcription factors, such as the tumor suppressor p53 [43] and ERα [44], GATA1 methylation by SET7 was not found in our study. The fact that knockdown of GATA1 decreases H3K4me, SET7, Pol II and TFIIB recruitment on VEGF promoter suggest that downregulation of VEGF expression by GATA1 knockdown is caused by both decreased recruitment of SET7-mediated H3K4me and disassociation of the basal transcriptional machinery containing Pol II and TFIIB on VEGF promoter.

Transcriptional regulation accounts for much of the up- and downregulation of VEGF in tumors [11, 12]. Sequence-specific transcription factors, such as early growth response protein 1 (Egr1) [45] and SP1 [13], can increase VEGF transcription in cancer cells. The proximal VEGF promoter regions include consensus binding sites for Egr1 (−77 to −70 bp) and SP1 (−88 to −66 bp). Here, we showed that GATA1 binds to the GATC sequence (−2 to +2 bp) containing the transcription start site (TSS) to enhance the transcription of VEGF in breast cancer cells. To the best of our knowledge, GATA1 is the first sequence-specific transcription factor that binds to the TSS of VEGF promoter. (Figure 7D). The GATA1 DNA-binding domain (DBD) consists of two zinc-finger domains located at the N and C termini, respectively [21, 22]. The C-terminal zinc-finger domain constitutes the primary DBD and is capable of binding to the consensus sequence GATA. The N-terminal zinc-finger domain binds independently to a related sequence containing a GATC core. Interestingly, we found that the C-terminal zinc-finger domain, but not the N-terminal zinc-finger domain, of GATA1 binds to the GATC motif.

GATA1 is essential for erythropoiesis, megakaryocyte maturation, and eosinophil production. In this study, we uncovered a novel function of GATA1 in regulating tumor angiogenesis and breast cancer cell growth *in vitro* and *in vivo*. Consistent with previous study [23, 24], we also found that GATA1 is overexpressed in breast cancer patients (data not shown). We further showed that, in breast cancer, GATA1 expression positively associates with tumor size, nodal status, grade, and GATA1 is an independent poor prognostic factor.

Very recently, SET7 has been shown to methylate β-catenin, which plays an important role in cell proliferation, cell fate determination, and tumorigenesis [46]. SET7 knockdown or the mutated β-catenin (K180R) that cannot be methylated increases the growth of human cervical cancer HeLa cell line *in vitro*. However, inactivation or ablation of SET7 causes G1/S cell cycle arrest upon DNA damage in lung cancer and osteosarcoma cells [47]. We showed that SET7 knockdown inhibits breast cancer cell growth *in vitro* and *in vivo*. Whether SET7 has tissue-specific roles in regulation of cancer cell growth remains to be investigated. In this study, we provide several lines of evidence showing that SET7 acts as an oncogene in breast cancer. First, SET7 knockdown decreases breast cancer cell growth both in culture and in nude mice. Second, in breast cancer patients, the expression of SET7 is upregulated and positively correlates with tumor size and nodal status. Third, like GATA1, SET7 is also an independent poor prognostic marker. Finally, decreased VEGF secretion in SET7 knockdown breast cancer cells reduces HUVEC cell proliferation, migration and tube formation as well as tumor angiogenesis. Since both GATA1 and SET7 are overexpressed in breast cancer patients, and GATA1 and SET7 promote not only breast cancer cell growth but also tumor angiogenesis, inhibition of GATA1 and/or SET7 may be a useful strategy for breast cancer therapy. Recently, SET7 inhibitors have been identified [48]. It will be interesting to investigate whether these inhibitors repress cancer cell growth and tumor angiogenesis.

## MATERIALS AND METHODS

### Plasmids, siRNAs and reagents

The eukaryotic expression vectors for FLAG-tagged GATA1 and SET7, and MYC-tagged GATA1 and SET7 were constructed by inserting PCR-amplified fragments into pcDNA3 (Invitrogen). Plasmids encoding GST or His fusion proteins were generated by cloning PCR-amplified sequences into pGEX-KG (Amersham Pharmacia Biotech) or pET-28a (Novagen). The VEGF promoter luciferase reporters were made by inserting PCR-amplified promoter fragments from genomic DNA into the pGL4-Basic vector (Promega). The cDNA target sequences of short hairpin RNAs (shRNAs) for GATA1 and SET7 are listed in Supplementary Table S4A. shRNAs were cloned into pSIH-H1-puro (System Biosciences) according to the manufacturers' protocols. Stable cell lines were generated by lentiviral transduction using pSIH-H1-puro (System Biosciences).

Anti-Myc (sc-40HRP) and anti-TFIIB (sc-225) antibodies were purchased from Santa Cruz Biotechnology; anti-Flag (A8592), anti-Flag M2 agarose (A2220), anti-SET7 (SAB1306218), and anti-GAPDH (G9295) were obtained from Sigma-Aldrich; anti-RNA Pol II (17–620), anti-H3K4me2 (17–677), anti-H3K4me3 (17–678), and anti-GATA1 (MABD167) were from Millipore; anti-H3K4me (ab8895), Anti-methylated lysine (ab23366), anti-CD31 (ab28364), and anti-SET7 (ab14820) were purchased from Abcam; anti-GATA1 (10917-2-AP) were purchased from Proteintech; anti-SET1 (A300–289A) and anti-MLL1 (A300–374A) were purchased from Bethyl. anti-GST (RPN1236) and anti-His (27471001) antibodies were purchased from GE Healthcare Life Sciences; and anti-VEGF (AF-293-NA) and anti-VEGF (MAB293-500) were purchased from R & D Systems.

### Cell culture, transfection, and luciferase reporter assay

The 293T embryonic kidney cells and ZR75-1, MCF7 and MDA-MB-231 breast cancer cells were routinely cultured in DMEM (Invitrogen) containing 10% FBS (Hyclone). The HUVEC cells were purchased from ATCC (American Type Culture Collection) and cultured in DMEM (Invitrogen) containing 10% FBS. Lipofectamine 2000 reagent was used for transfections following the manufacturer's protocol (Invitrogen). Lentiviruses were produced by cotransfection of 293 T cells with recombinant lentivirus vectors and pPACK Packaging Plasmid Mix (System Biosciences) using Megatran reagent (Origene). Lentiviruses were collected 48 hours after transfection and added to the medium of target cells with 8 μg/ml polybrene (Sigma-Aldrich). Stable cell lines were selected in 1 μg/ml puromycin for approximately 2 months. Pooled clones or individual clones were screened by standard immunoblot protocols and produced similar results. Luciferase reporter assays were performed as described previously [50].

### Screening for transcription factors that regulate VEGF promoter luciferase activity

A high-throughput assay based on reverse transfection of 704 transfection-ready cDNA plasmids from Transcription factor GFC-Transfection Array was used according to the manufacturers' instructions (Origene). Briefly, the Turbofectin 8.0 reagent (Origene), 100 ng of the VEGF-Luc reporter vector, and 100 ng of β-galactosidase reporter were added to each well of 384-well plates containing 60 ng of distinct cDNA plasmids. Complex formation was allowed for 20 min at room temperature before the addition of ZR75-1 cells (7500 cells/well). After a 48 h incubation, cells were harvested and analyzed for luciferase and β-galactosidase activities as described previously [50].

### Real-time reverse transcription-PCR (RT-PCR)

Total RNA was extracted using TRIzol reagent according to the manufacturer's protocol (Invitrogen). RNA was reverse transcribed into cDNA by Quantscript RT Kit (Tiangen). The relative expression of GATA1, SET7, or VEGF was measured by real-time PCR with SYBR-green dye. The primers used for the GATA1, SET7, and VEGF gene products are listed in Supplementary Table S4B.

### Enzyme-linked immunosorbent assay (ELISA) for human VEGF protein expression

Cells were seeded into 12-well plates. Forty eight hours later, cell supernatant was harvested to measure the concentration of VEGF_165_ using human VEGF kit (R & D systems) according to the manufacturer's instructions. The concentration of VEGF was normalized for total protein concentration.

### Wound healing assay

The confluent cell monolayer in a 12-well plate was wounded by manually scraping the cells with a white pipette tip. The cells were treated with conditioned medium. Cell migration into the wound surface was monitored at various times. Quantitation was done by measuring the distance of the wound edge of the migrating cells from the start point to the migrated point in three independent experiments.

### Tube formation assay

Thawed extracellular matrix (ECM) gel solution was added to the well of a pre-chilled 96-well sterile plate, and was incubated 30 min to 1 h at 37°C to allow the ECM solution to form a gel. 1∼2 × 10^5^ HUVEC cells/ml endothelial cells were suspended in the medium containing 0.5% serum and conditioned medium. The HUVEC cell suspension (1.5∼3 × 10^4^ cells) was added onto the solidified ECM gel per well. The plate was incubated at 37°C for 4 to 18 h. Then tube formation in each well was monitored and photographed using an inverted microscope. The tube length was measured using Image-Pro Plus.

### Chick chorioallantoic membrane (CAM) assay

Fertilized eggs were incubated at 37°C and 60% humidity for 10 days. A square window was made on the air sac to expose the CAM. Sterile 0.25 cm-diameter filter papers were applied onto the surfaces of the CAM, and 100 μl of conditioned medium was added to the filter immediately. The windows were sealed, and the eggs were incubated for another 3 days. The CAMs were fixed with methanol: acetone (1:1, v/v) for 15 min and were photographed by Nikon D7000 camera (Nikon, Japan), and the number of blood vessels around the filter papers within 1 mm was counted.

### GST pull-down and coimmunoprecipitation assays

For GST pull-down assay, GST or His fusion proteins were expressed and purified according to the manufacturers' instructions (Amersham Pharmacia and Qiagen). Purified His fusion proteins were incubated with GST fusion protein bound to GST beads for 4 h at 4°C. After washing, the precipitated components were analyzed by immunoblot. For coimmunoprecipitation assay, cells were harvested and lysed in lysis buffer on ice for 30 min. After centrifugation at 4°C at 12,000 rpm for 15 min, antibodies were added to the supernatant and rolled at 4°C overnight. Protein G or A Agarose (Santa Cruz) was then added to the samples, and the samples were rolled at 4°C for 2 h. After the beads were washed three times with lysis buffer, the pellets were dissolved into 2 × SDS loading buffer after centrifugation and boiled at 100°C for 10 min. Proteins were analyzed by immunoblot with indicated antibodies.

### Chromatin immunoprecipitation (ChIP) and re-ChIP

ChIP assay was performed using the Magna ChIP Assay Kit (Millipore) according to the manufacturer's instructions. For re-ChIP, complexes were eluted from the primary immunoprecipitation by incubation with 10 mM DTT at 37°C for 30 min and diluted 1:50 in re-ChIP buffer (150 mM NaCl, 1% Triton X-100, 2 mM EDTA, 20 mM Tris-HCl, pH 8.1) followed by re-immunoprecipitation with the second antibodies. Real-time PCR was performed to detect relative occupancy. The primers used for real-time PCR are listed in Supplementary Table S4C.

### Electrophoretic mobility shift assay (EMSA)

DNA-protein binging assays were carried out with nuclear extract using LightShift Chemiluminescent EMSA Kit according to the manufacturer's instructions (Pierce). Synthetic complementary oligonucleotides were 5′-biotinylated and annealed for 2 h at room temperature. The sequences of the oligonucleotides used are listed in Supplementary Table S4D. After the biotin-labeled DNA was incubated with the nuclear extracts, the DNA-protein complexes were subjected to a 5% native polyacrylamide gel electrophoresis and transferred to a nylon membrane. The membrane was immediately cross-linked at 120 mJ/cm^2^ for 1 min using a UV-light cross-linker instrument equipped with 254 nm bulbs. The bands was detected with the kit.

### Animal experiments

Animal studies were performed in accordance with protocols approved by the Institutional Animal Care and Use Committee at Beijing Institute of Biotechnology. Five million breast cancer cells were injected into the abdominal mammary fat pad of 6-week-old female nude mice. Tumor size was measured at indicated times using calipers. Tumor volume was calculated according to the following formula: volume = (longest diametershortest diameter^2^)/2.

### Clinical samples and immunohistochemistry

Eighty cases of primary breast carcinomas and adjacent noncancerous tissues were obtained from Chinese PLA General Hospital, with the informed consent of patients and with the approval of the Institutional Review Committees of Chinese PLA General Hospital. All cases were female with 25–71 years of age (mean age: 48.3 years). Breast tissue samples were obtained during surgery without any other treatment. The histological types were defined according to the WHO criteria, and the staging was based on the 2010 TNM classification. Breast cancer subtypes were determined as following definitions: Luminal A (ER/PR+, Her2−), Ki-67 low (< 14%); Luminal B (ER/PR+, Her2−, Ki-67 high and ER/PR+, Her2−, any Ki-67); Her2 overexpression (ER−, PR−, Her2+); basal-like (ER−, PR−, Her2−). The patients got similar treatments based on the subtypes. The detailed information of breast cancer specimens was demonstrated in Supplementary Table S3.

Immunohistochemistry (IHC) of formalin-fixed paraffin-embedded samples was performed as described previously [50]. Rabbit anti-GATA1 (10917-2-AP, Proteintech), rabbit anti-SET7 antibody (SAB1306218, Sigma-Aldrich), mouse anti-VEGF (MAB293–500, R & D Systems), and rabbit anti-CD31 antibody (ab28364, Abcam) were used at dilutions of 1: 100, 1:200, 1:100 and 1:50 as the primary antibodies for IHC. GATA1, SET7 or VEGF score was generated by multiplying the percentage of stained cells (0–25%, 1+; 26%–50%, 2+; 51–75%, 3+; 76%–100%, 4+) by the intensity of the staining (low, 1+; medium, 2+, strong, 3+). We defined score ≤ 6 as low GATA1, SET7, or VEGF, and score > 6 as high GATA1, SET7, or VEGF.

### Statistical analysis

Statistical significance in the preclinical experiments was assessed by two-tailed Student's *t* test. The correlation between GATA1 or SET7 expression and clinicopathologic characteristics was determined using Pearson's *χ2* test. Estimation of disease-free survival and overall survival was performed using the Kaplan-Meier method, and differences between survival curves were examined with the log-rank test. The Cox regression model was used to perform univariate and multivariate analyses. All statistical tests were two-sided. Statistical calculations were performed using SPSS 17.0. In all assays, *P* < 0.05 was considered statistically significant.

## SUPPLEMENTARY MATERIALS TABLES AND FIGURES


