# Supplementary material for: The transcription factor GATA1 and the histone methyltransferase SET7 interact to promote VEGF-mediated angiogenesis and tumor growth and predict clinical outcome of breast cancer
Source: Oncotarget. 2016 Feb 2;7(9):9859–75. doi: 10.18632/oncotarget.7126 (PMC4891089; doi:10.18632/oncotarget.7126)
Supplement: Supplementary file 1 [file oncotarget-07-09859-s001.pdf]

The transcription factor GATA1 and the histone methyltransferase SET7 interact to promote VEGF-mediated angiogenesis and tumor growth and predict clinical outcome of breast cancer

Supplementary Materials

Supplementary Table S1: Cox univariate and multivariate analysis of disease-free survival and overall survival in patients with breast cancer

Factor	Univariate			Multivariate		
	P value	HR	95% CI	*P* value	HR	95% CI
Disease-free survival						
Tumor size: ≤ 20 vs > 20 mm	4.6×10^{-5}	3.442	1.905−6.711	0.002	2.768	1.823−5.457
Lymph node: negative vs positive	0.001	2.917	1.867−5.791	0.005	2.226	1.349−4.887
Grade: I vs II vs III	0.002	2.936	1.878−5.846	0.039	1.745	1.086−3.652
ER: negative vs positive	0.0001	0.165	0.067−0.405	0.264	0.508	0.263−1.239
PR: negative vs positive	0.027	0.388	0.168−0.698	0.384	0.612	0.304−1.428
HER2: negative vs positive	0.069	1.420	0.946−3.497	0.198	1.354	0.891−2.365
GATA1: negative vs positive	0.001	2.916	1.847−5.700	0.003	2.512	1.635−5.186
SET7: negative vs positive	0.001	2.908	1.821−5.688	0.024	1.824	1.128−3.941
Overall survival						
Tumor size: ≤ 20 vs > 20 mm	0.0002	6.523	3.423−10.45	0.002	5.041	2.215−9.584
Lymph node: negative vs positive	0.001	5.127	2.321−9.886	0.008	4.256	1.628−7.852
Grade: I vs II vs III	0.0007	5.786	2.845−10.06	0.026	3.014	1.565−6.952
ER: negative vs positive	0.0002	0.143	0.051−0.424	0.042	0.428	0.256−0.907
PR: negative vs positive	0.133	0.597	0.400−1.338	0.151	0.648	0.463−1.586
HER2: negative vs positive	0.425	1.143	0.742−2.076	0.574	1.135	0.693−1.983
GATA1: negative vs positive	0.005	4.188	1.887−7.839	0.027	2.941	1.504−6.548
SET7: negative vs positive	0.006	4.059	1.727−7.479	0.042	2.524	1.394−5.412

Supplementary Table S2: Correlation of GATA1 and SET7 status with clinical factors*

Clinical characteristics	Total cases	GATA1 low	GATA1 high	*P* value	SET7 low	SET7 high	*P* value
Age				0.238			0.205
≤ 50 years	48	19	29		23	25	
> 50 years	32	17	15		20	12	
Tumor size				0.001			0.001
≤ 20 mm	42	26	16		30	12	
> 20 mm	38	10	28		13	25	
Nodal status				0.004			0.016
Negative	46	27	19		30	16	
Positive	34	9	25		13	21	
Grade				0.001			0.002
I	18	12	6		13	5	
II	37	19	18		23	14	
III	25	5	20		7	18	
ERα				0.049			0.033
Negative	27	8	19		10	17	
Positive	53	28	25		33	20	
PR				0.178			0.226
Negative	31	11	20		14	17	
Positive	49	25	24		29	20	
HER2				0.609			0.899
Negative	60	28	32		32	28	
Positive	20	8	12		11	9	

**P* values were assessed by Pearson chi-square test.

Supplementary Table S3: Baseline patient characteristics

Characteristic	No.	%
Age		
≤ 50 years	48	60.0
> 50 years	32	40.0
Stage		
I	18	22.5
II	41	51.3
III	21	26.2
Nodal status		
Negative	46	57.5
Positive	34	42.5
Grade		
I	18	22.5
II	37	46.3
III	25	31.2
ER		
Negative	27	33.8
Positive	53	66.2
PR		
Negative	31	38.8
Positive	49	61.2
HER2		
Negative	60	75.0
Positive	20	25.0
Subtype		
Luminal A	38	47.5
Luminal B	22	27.5
HER2 overexpression	8	10.0
Basal-like	12	15.0

Supplementary Table S4: Sequences for shRNAs, RT-PCR, ChIP and EMSA

Supplementary Table S4A: The cDNA target sequences of shRNAs

Gene	Target sequence (5′ → 3′)
GATA1–1	TAGTGCTTATGGGGGCCCTGACTTT
GATA1–2	GAAGCGCCTGATTGTCAGTAAACGG
SET7–1	GGGCACCTGGACGATGACGGA
SET7–2	GCCTTGTAGGAGAAGTAAA
VEGFR2	TAAACAGGAGGAGAGCTCAGTGTGG

Supplementary Table S4B: Primers used for real-time RT-PCR

Gene	Forward (5′ → 3′)	Reverse (5′ → 3′)
GATA1	GGAGACTTTGAAGACAGAG	GGAGAGGAATAGGCTGCTG
SET7	CGAATTACACACCAAGAGGTT	TAGGCAACGGTGAGCTCTTC
$VEGF_{121}$	ATAGAGCAAGACAAGAAAAATG	ATCGTTCTGTATCAGTCTTTCCT
$VEGF_{165}$	AGAGCAAGACAAGAAAATCC	TACAAACAAATGCTTTCTCC
bcl-xL	GAGCCTTGGATCCAGGAGAA	CATGCCCGTCAGGAACCAGC
Myb	TGTTCTCAAAGCATTTACAGTACC	GATGTCATCTGCTCCTCCATC
Myc	GGCTCTCCTTGCAGCTGCTT	TCGTCGCAGTAGAAATACGG

Supplementary Table S4C: Primers used for ChIP

VEGF Promoter	Forward (5′ → 3′)	Reverse (5′ →3 ′)
−23~+43	AAGTCGGCTGGTAGCGGG	CGCCACGACCTCCGAGCTA
−1241~−1174	CAGAAGATGAGCTATGAGTCT	AGAGCTCCCACCAGGCCA
−206~−114	GGTGGGGGTCGAGCTTCC	CCTTCCACACGCGGCTCG
−113~−6	GCTGAGGCTCGCCTGTCC	CCCGCTACCAGCCGACTT
+49~+109	GGCTAGCACCAGCGCTCTG	GTGAGTCCGCTGACCGGTC
+132~+244	AGGGCGCTCGGTGCTGGA	CAAGTGGGGAATGGCAAGCA
+295~+349	TACTTCCCCAAATCACTGTGG	CTCTGGAGCTCTTGCTACCTC

Supplementary Table S4D: Sequences of the oligonucleotides used for EMSA

Probe	Forward (5′ → 3′)	Reverse (5′ → 3′)
GATC	TGGTAGCGGGGAGGATCGCGGAGGCTTGGG	CCCAAGCCTCCGCGATCCTCCCCGCTACCA
GATC-M	TGGTAGCGGGGAGTCGAGCGGAGGCTTGGG	CCCAAGCCTCCGCTCGACTCCCCGCTACCA
GATA	CTGGGCTTGGGCTGATAGAAGCCTTGGCCC	GGGCCAAGGCTTCTATCAGCCCAAGCCCAG

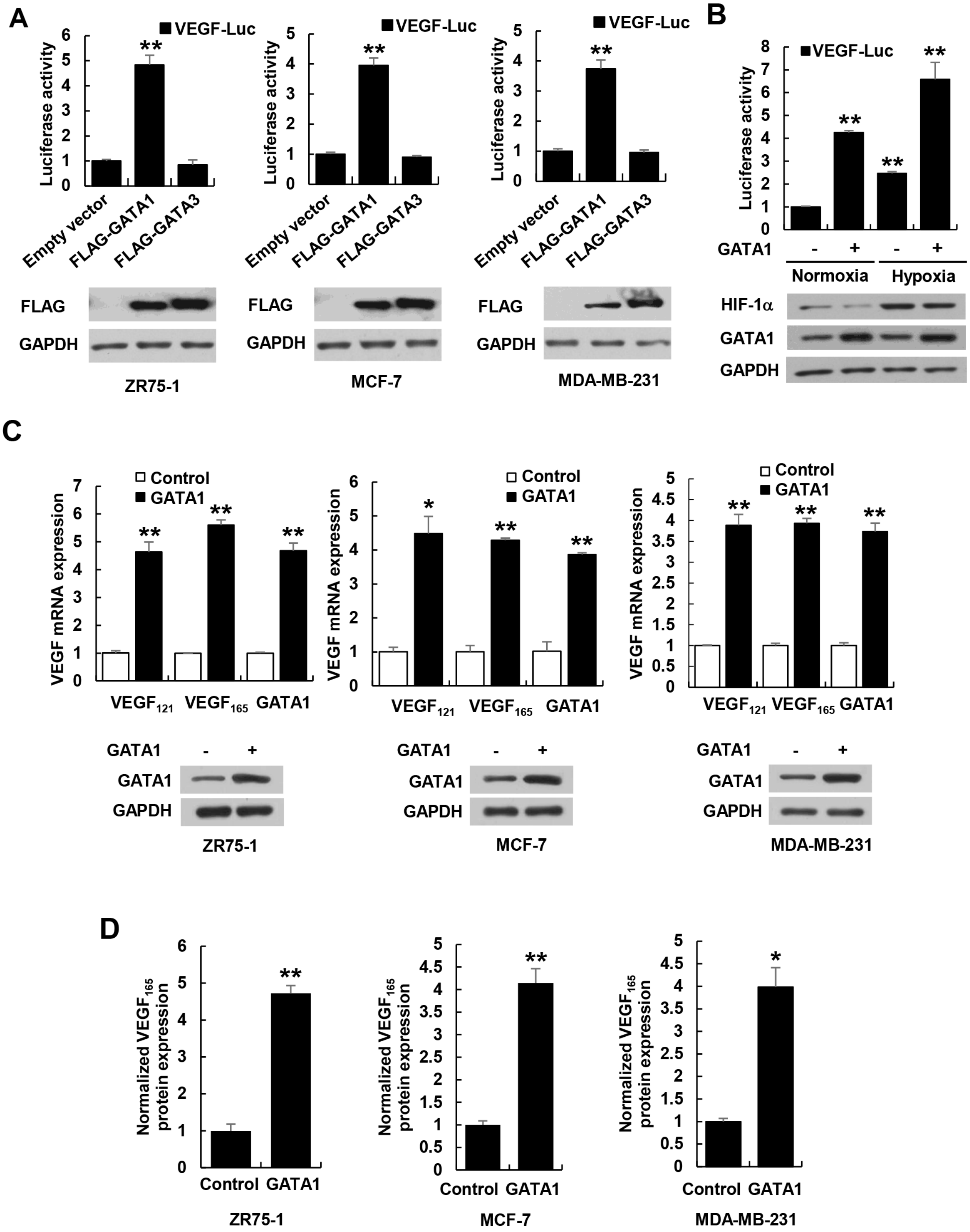

Supplementary Figure S1: GATA1 modulates VEGF expression in breast cancer cells. (**A**) Luciferase reporter assays in ZR75-1, MCF-7 and MDA-MB-231 cells cotransfected with VEGF-Luc and GATA1, GATA3 or empty vector (control). Representative Western blot shows the expression of GATA1 and GATA3. GAPDH was used as a loading control. (**B**) Luciferase reporter assays in MCF7 cells cotransfected with VEGF-Luc and GATA1 or empty vector (control) under normoxic or hypoxic (1% O_2) conditions. Representative Western blot shows the expression of HIF-1α and GATA1. (**C**) Real-time RT-PCR analyses of $VEGF_{121}$ and $VEGF_{165}$ expression in ZR75-1, MCF-7 and MDA-MB-231 cells infected with lentivirus carrying GATA1 or empty vector (control). Representative Western blot indicates the expression of GATA1. (**D**) VEGF concentration in cell supernatants from ZR75-1, MCF7 and MDA-MB-231 cells infected as in (C). All values shown are mean ± SD of triplicate measurements that have been repeated 3 times with similar results. *$P < 0.05$, **$P < 0.01$ versus empty vector.

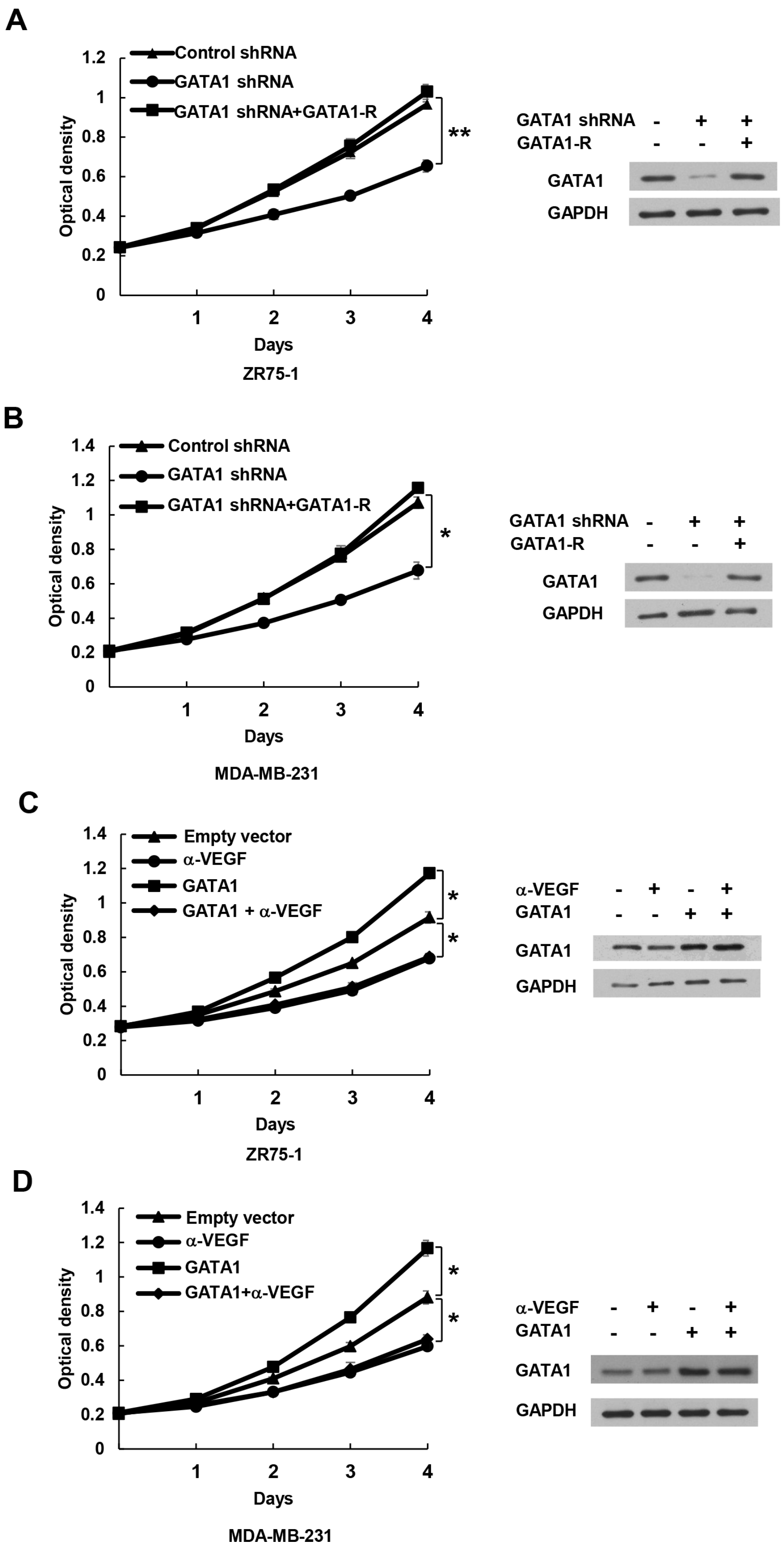

Supplementary Figure S2: Cancer cell-secreted VEGF regulated by GATA1 controls HUVEC proliferation. (**A**, **B**) Cell proliferation assays in HUVEC cells cultured in conditioned medium from ZR75-1 (A) and MDA-MB-231 (B) cells stably infected with GATA1 shRNA or GATA1 shRNA plus shRNA-resistant GATA1 (GATA1-R). Representative Western blot shows GATA1 expression. (**C**, **D**) Cell proliferation assays in HUVEC cells cultured in conditioned medium from ZR75-1 (C) and MDA-MB-231(D) stably infected with GATA1 and treated with a VEGF neutralizing antibody (α-VEGF). Representative Western blot shows the expression of GATA1. All values shown are mean ± SD of triplicate measurements that have been repeated 3 times with similar results. $^*P < 0.05$, $^{**}P < 0.01$.

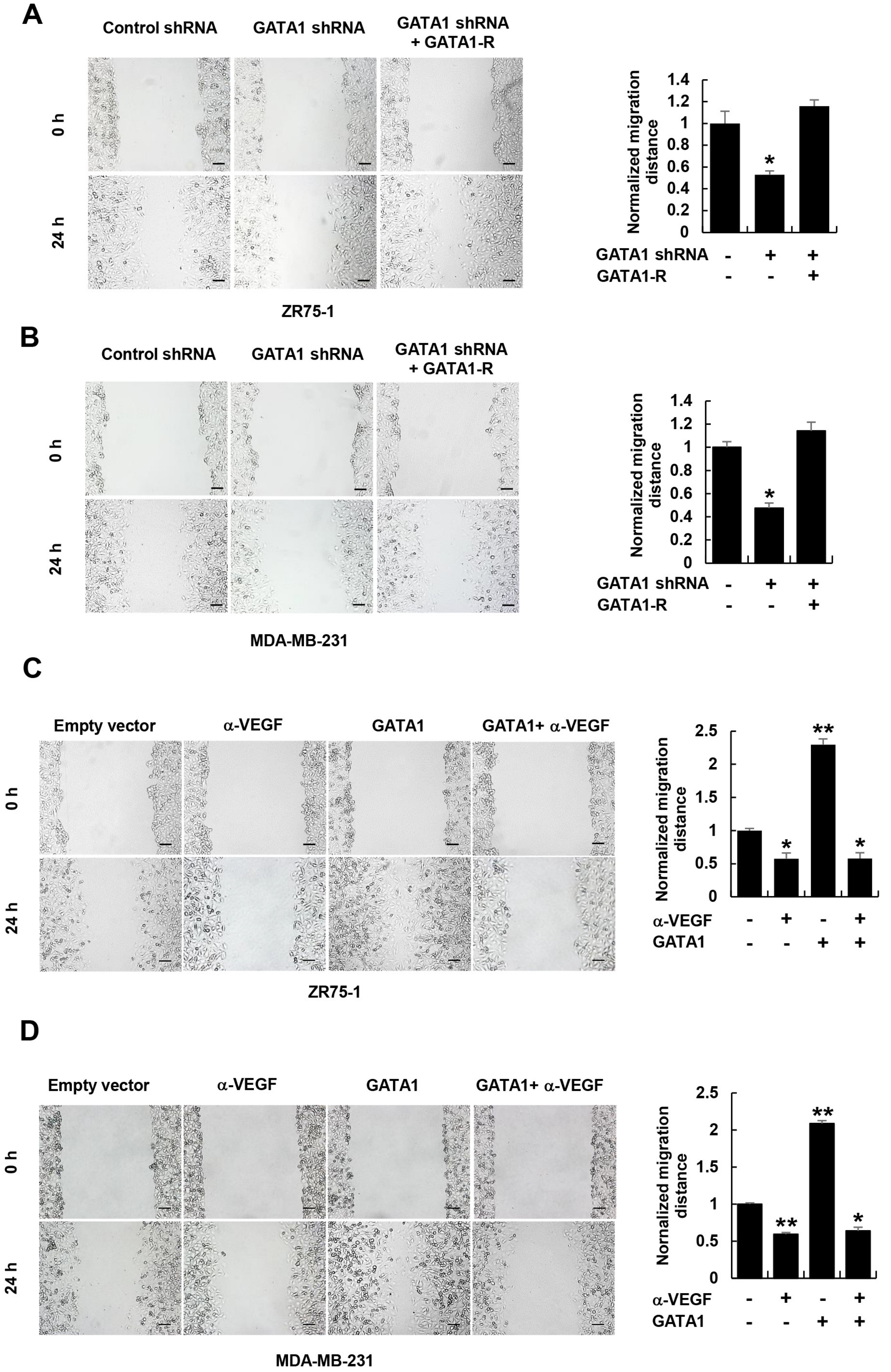

Supplementary Figure S3: Cancer cell-secreted VEGF regulated by GATA1 modulates HUVEC migration. (**A**, **B**) Wound healing assays for HUVEC cells cultured in conditioned medium from ZR75-1 (A) and MDA-MB-231 (B) cells stably infected with GATA1 shRNA or GATA1 shRNA plus GATA1-R. Scale bar: 100 μm. (**C**, **D**) Wound healing assays in HUVEC cells cultured in conditioned medium from ZR75-1 (C) and MDA-MB-231 (D) stably infected with GATA1 and treated with a VEGF neutralizing antibody. Scale bar: 100 μm. All values shown are mean ± SD of triplicate measurements that have been repeated 3 times with similar results. *$P < 0.05$ versus control shRNA. *$P < 0.05$, **$P < 0.01$ versus empty vector.

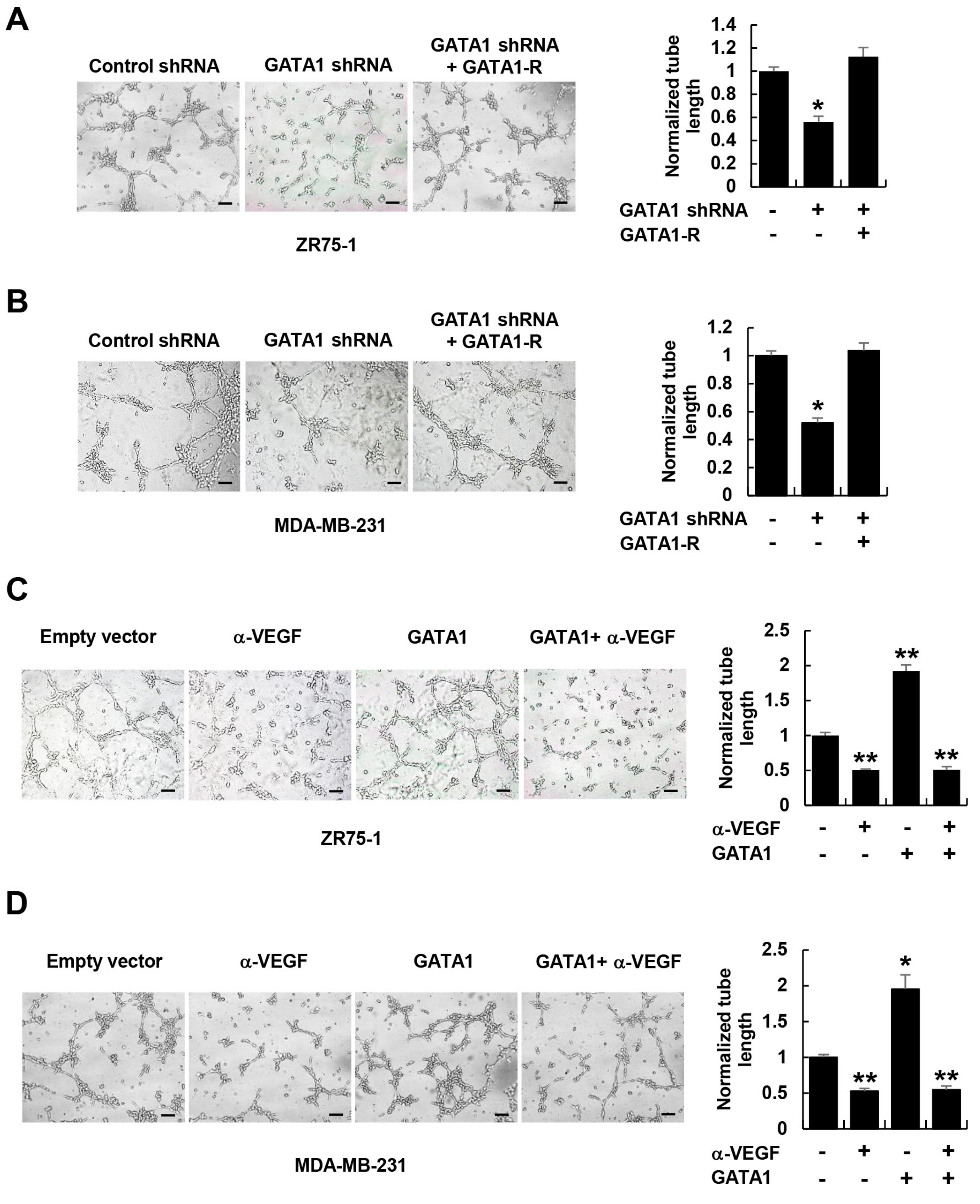

Supplementary Figure S4: Cancer cell-secreted VEGF regulated by GATA1 is required for HUVEC tube formation. (**A**, **B**) Tube formation assays for HUVEC cells cultured in conditioned medium from ZR75-1 (A) and MDA-MB-231 (B) cells stably infected with GATA1 shRNA or GATA1 shRNA plus GATA1-R. Scale bar: 100 μm. (**C**, **D**) Tube formation assays for HUVEC cells cultured in conditioned medium from ZR75-1 (C) and MDA-MB-231 (D) cells stably infected with GATA1 and treated with α-VEGF. Scale bar: 100 μm. Data shown are mean ± SD of triplicate measurements that have been repeated 3 times with similar results. **P* < 0.05, ***P* < 0.01 versus control shRNA or empty vector.

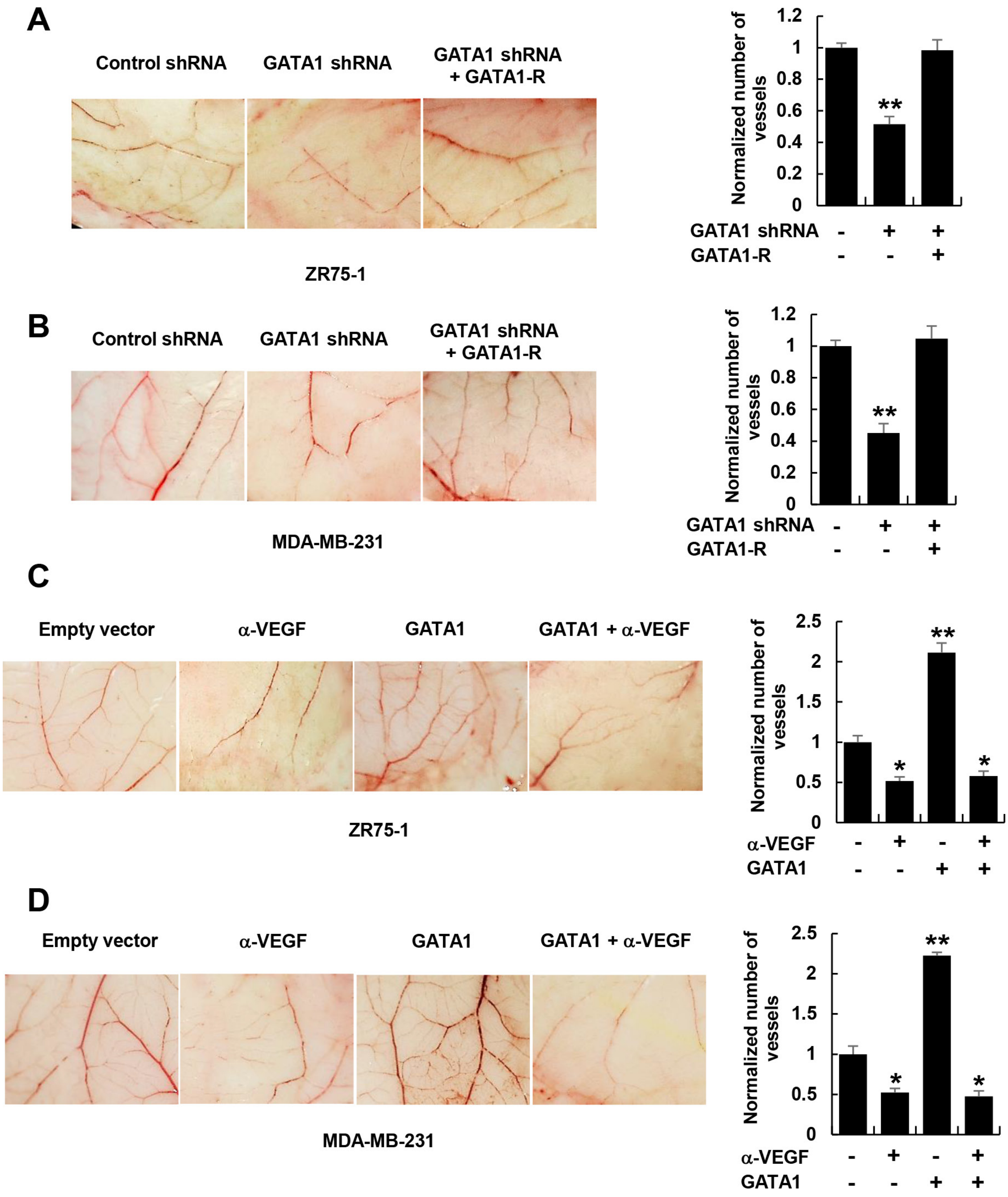

Supplementary Figure S5: Cancer cell-secreted VEGF regulated by GATA1 is responsible for angiogenesis. (**A**, **B**) CAM assays with conditioned medium from ZR75-1 (A) and MDA-MB-231 (B) cells stably infected with GATA1 shRNA or GATA1 shRNA plus GATA1-R. (**C**, **D**) CAM assays with conditioned medium from ZR75-1 (C) and MDA-MB-231 (D) cells stably infected with GATA1 and treated with α-VEGF. All values shown are mean ± SD of triplicate measurements and have been repeated 3 times with similar results (A–D). *$P < 0.05$, **$P < 0.01$ versus control shRNA or empty vector.

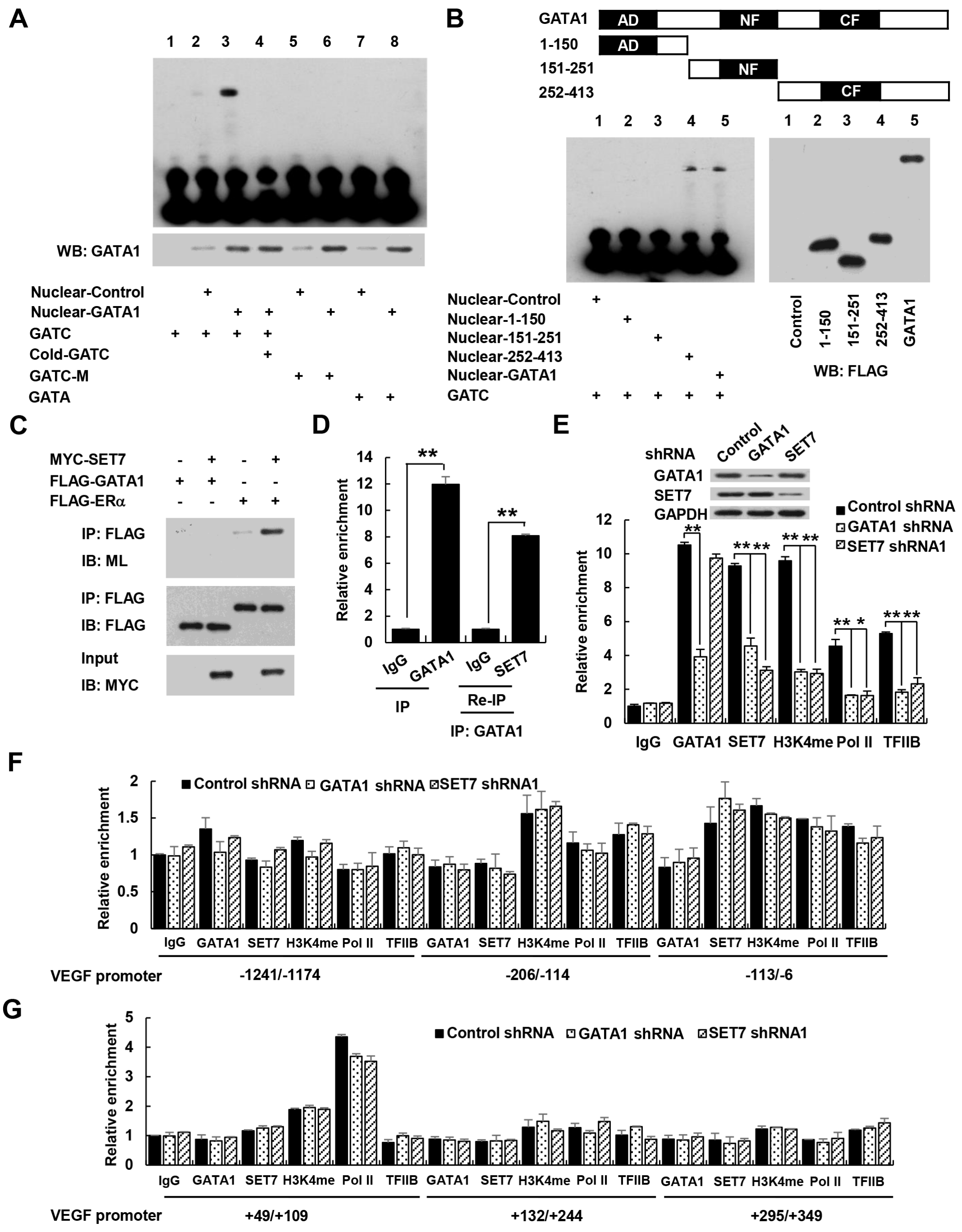

Supplementary Figure S6: GATA1 binds to the GATC site of VEGF promoter and recruits SET7, TFIIB and RNA polymerase II to the VEGF promoter. (**A**) EMSA assay of GATA1 binding site in the VEGF promoter. Nuclear extracts from GATA1 knockdown MCF7 cells transfected with empty vector or FLAG-tagged GATA1 were incubated with different biotin-labelled probes containing the GATA or GATC site or mutated GATC site (GATC-M) of the VEGF promoter as indicated. For competition experiments, a 100-fold molar excess of unlabeled GATC was mixed with the biotin-labelled probe. Western blot shows the expression of GATA1 with anti-GATA1. (**B**) EMSA assay of the GATA1 domain that binds to the VEGF promoter. Nuclear extracts from GATA1 knockdown MCF7 cells transfected with empty vector (control), FLAG-tagged GATA1 or FLAG-tagged GATA1 deletion mutants were incubated with biotin-labelled probes containing the GATC site, as indicated. Western blot shows the expression of GATA1 and its deletion mutants with anti-FLAG. AD, activation domain; NF, N-terminal zinc finger; CF, C-terminal zinc finger. (**C**) SET7 does not methylate GATA1. Cell lysates from MCF7 cells transfected with MYC-tagged SET7 and FLAG-tagged GATA1 or ERα were immunoprecipitated (IP) with FLAG antibodies, followed by immunoblotting (IB) with the indicated antibodies. ML, anti-methylated lysine. (**D**) Re-ChIP analysis of the occupancy of GATA1 and SET7 on VEGF promoter (−23/+43) in MDA-MB-231 cells. (**E**) ChIP analysis of MDA-MB-231 cells stably infected with lentivirus carrying GATA1 shRNA or SET7 shRNA1 on VEGF promoter (−23/+43) with the indicated antibodies. Western blot shows the knockdown effects of GATA1 and SET7. Data shown are mean ± SD of triplicate measurements and have been repeated 3 times with similar results (D, E). **P* < 0.05, ***P* < 0.01. (**F**, **G**) ChIP analysis of MCF7 cells stably as infected as in Figure. 4F on the indicated regions of the VEGF promoter with the indicated antibodies.

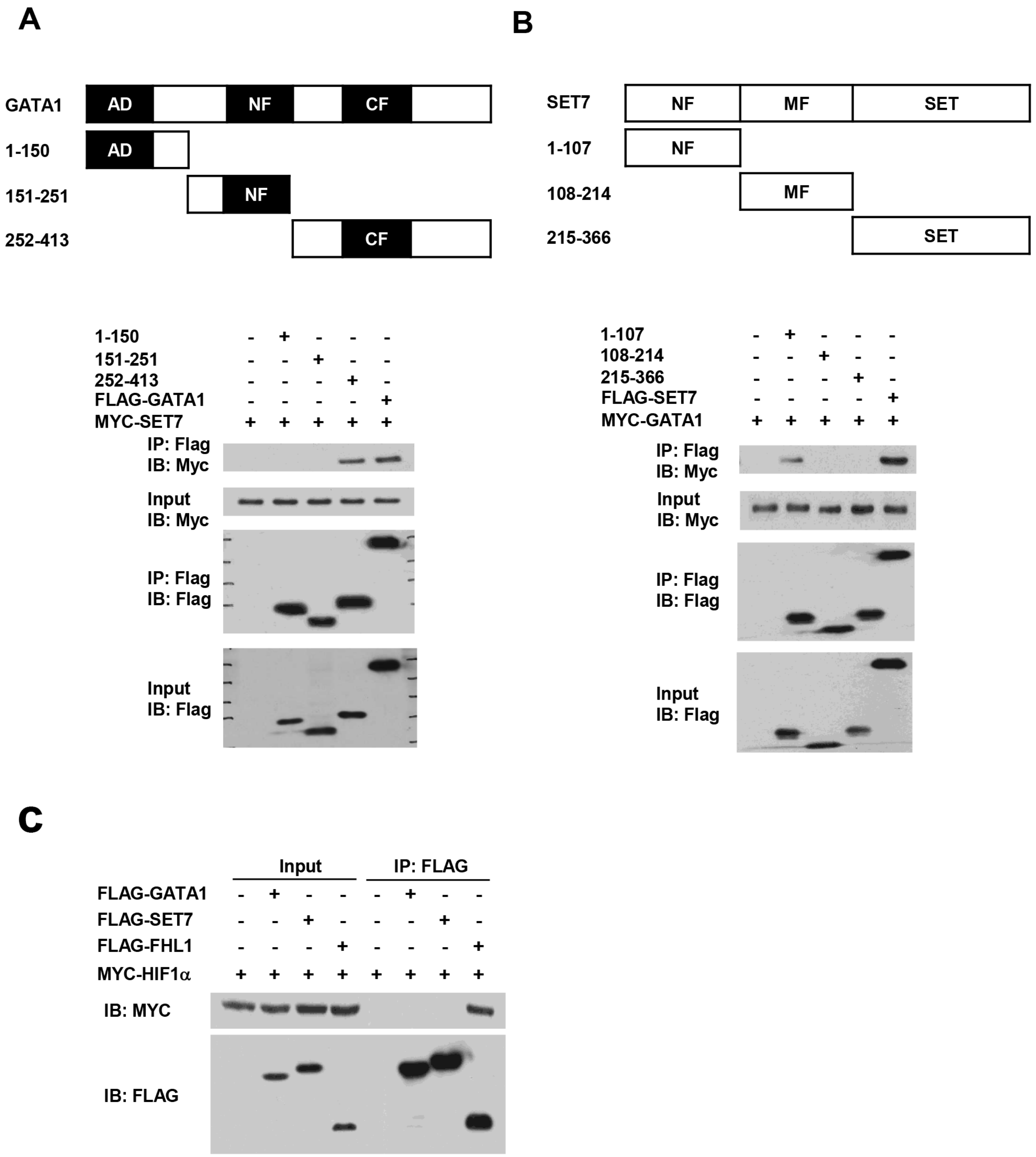

Supplementary Figure S7: Mapping of the GATA1 and SET7 interaction domains. (**A**) Mapping of interaction region of SET7 in GATA1. HEK293T cells were cotransfected with MYC-tagged SET7 and FLAG-tagged GATA1 or its deletion mutants. Cell lysates were immunoprecipitated with anti-FLAG, followed by immunoblotting with the indicated antibodies. Schematic diagram of GATA1 and its deletion mutants is shown. AD, activation domain; NF, N-terminal zinc finger; CF, C-terminal zinc finger. (**B**) Mapping of the GATA1 interaction region in SET7. HEK293T cells were cotransfected with MYC-tagged GATA1 and FLAG-tagged SET7 or its deletion mutants. Cell lysates were immunoprecipitated and analyzed as in (A). Schematic diagram of SET7 and its deletion mutants is shown. NF, N-terminal fragment; MF, middle region fragment; SET, SET domain-containing fragment. (**C**) Interaction of GATA1, SET7, and FHL1 with HIF-1α. HEK293T cells were cotransfected with MYC-tagged HIF-1α and FLAG-tagged GATA1, SET7 or FHL1. Cell lysates were immunoprecipitated with anti-FLAG, followed by immunoblotting with the indicated antibodies.

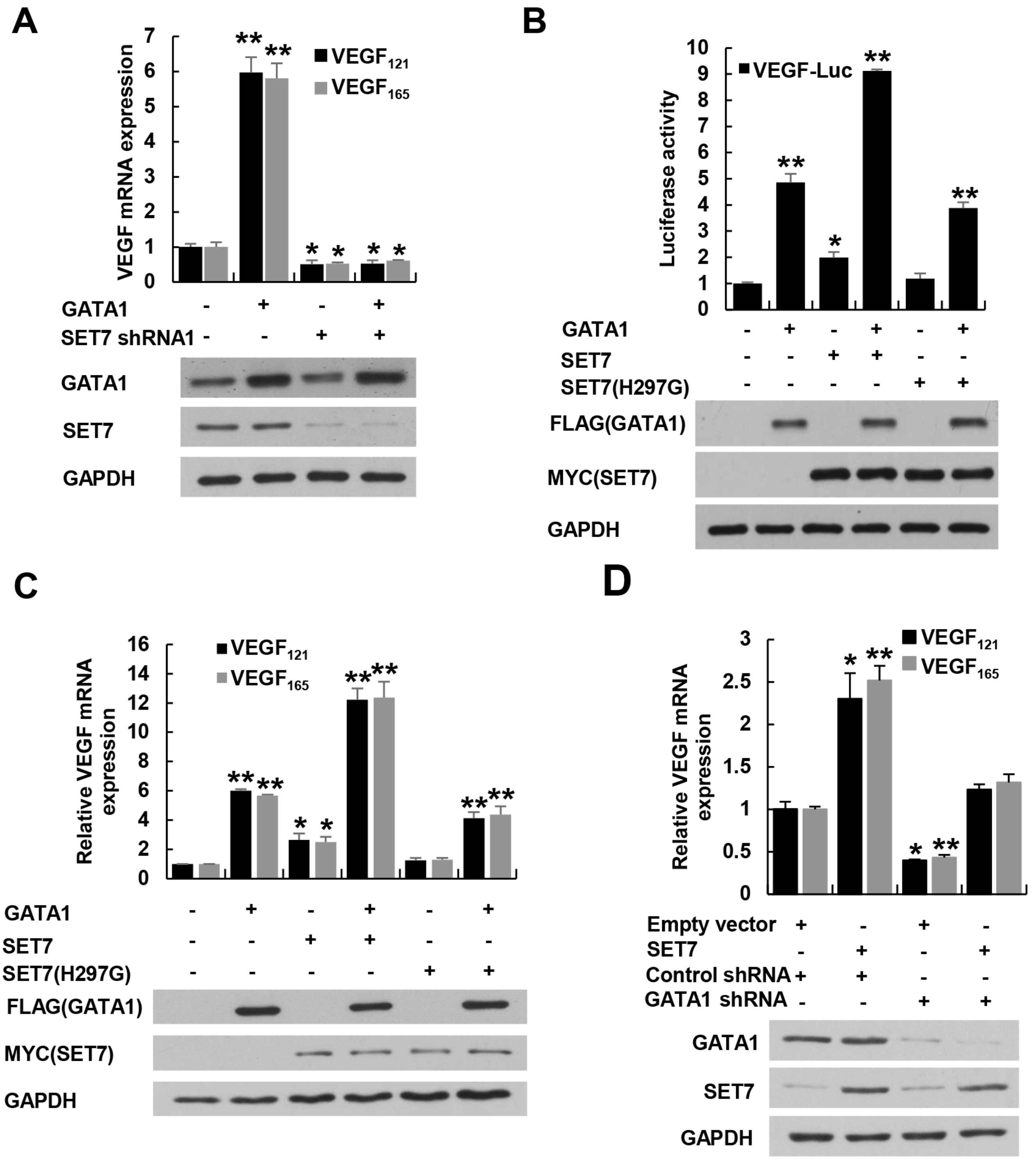

Supplementary Figure S8: GATA1 regulates VEGF expression through SET7. (**A**) Real-time RT-PCR analysis of $VEGF_{121}$ and $VEGF_{165}$ expression in MDA-MB-231 cells infected with lentiviruses carrying GATA1 or empty vector and SET7 shRNA1 or control shRNA. Western blot shows the expression of GATA1 and SET7. (**B**) Luciferase reporter assays in MCF7 cells cotransfected with VEGF-Luc, GATA1 and either SET7 or methyltransferase-deficient SET7 (H297G). Cell lysates were detected by Western blot with the indicated antibodies. (**C**) Real-time RT-PCR analysis of $VEGF_{121}$ and $VEGF_{165}$ expression in MCF7 cells cotransfected with GATA1 and either SET7 or SET7 (H297G). (**D**) Real-time RT-PCR analysis of $VEGF_{121}$ and $VEGF_{165}$ expression in MCF7 cells infected with lentiviruses carrying SET7 or empty vector and Control shRNA or GATA1 shRNA. Western blot shows the expression of GATA1 and SET7. All values shown are mean ± SD of triplicate measurements and have been repeated 3 times with similar results. $*P < 0.05$, $**P < 0.01$ versus corresponding control.

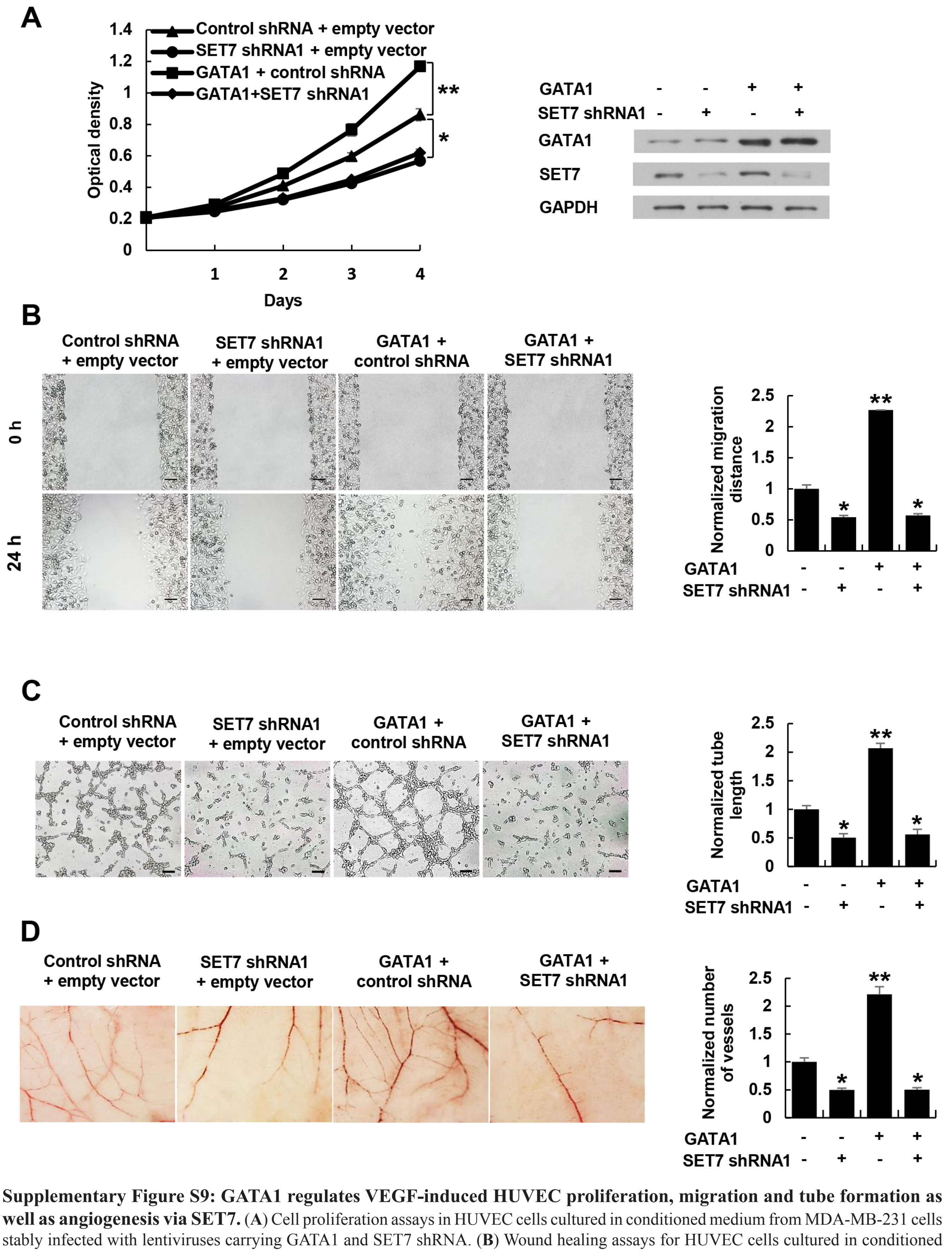

Supplementary Figure S9: GATA1 regulates VEGF-induced HUVEC proliferation, migration and tube formation as well as angiogenesis via SET7. (**A**) Cell proliferation assays in HUVEC cells cultured in conditioned medium from MDA-MB-231 cells stably infected with lentiviruses carrying GATA1 and SET7 shRNA. (**B**) Wound healing assays for HUVEC cells cultured in conditioned medium from MDA-MB-231 stably infected as in (A). Scale bar: 100 µm. (**C**) Tube formation assays for HUVEC cells cultured in conditioned medium from MDA-MB-231 cells stably infected as in (A). Scale bar: 100 µm. (**D**) CAM assays with conditioned medium from MDA-MB-231 cells stably infected as in (A). All values shown are mean ± SD of triplicate measurements and have been repeated 3 times with similar results. **P* < 0.05, ***P* < 0.01 versus corresponding control.

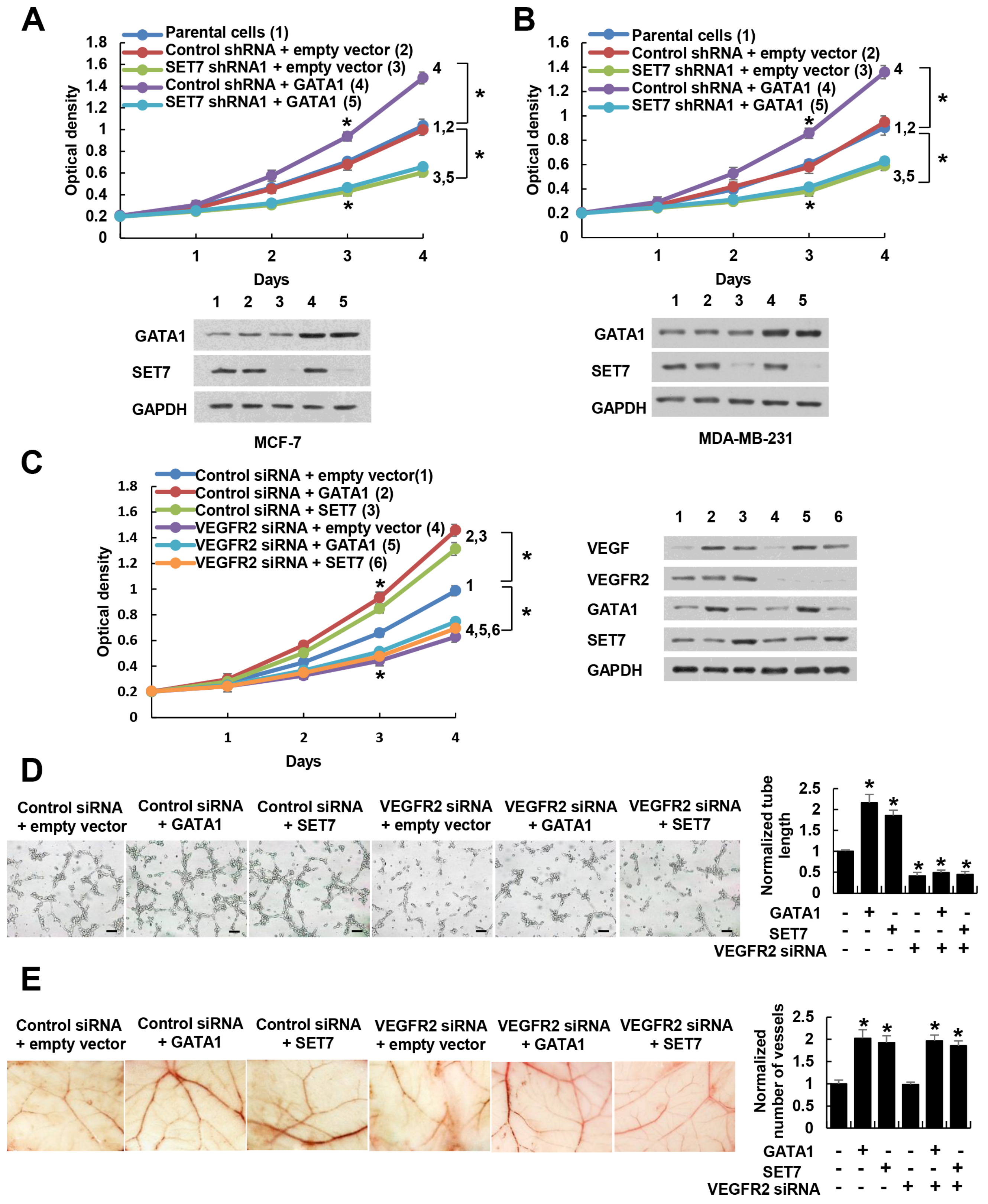

Supplementary Figure S10: GATA1 regulates breast cancer cell proliferation through SET7. (**A**, **B**) Cell proliferation assays in MCF7 (A) and MDA-MB-231 cells (B) stably infected with lentiviruses carrying the indicated constructs. Western blot shows the expression of GATA1 and SET7. All values shown are mean ± SD of triplicate measurements and have been repeated 3 times with similar results. *$P < 0.05$ on day 4. (**C**) Cell proliferation assays in MCF7 stably infected with lentiviruses carrying GATA1 or SET7 cells and transfected with control siRNA or VEGFR2 siRNA. Western blot shows the expression of VEGF, VEGFR2, GATA1 and SET7. *$P < 0.05$ on day 4. (**D**) Tube formation assays for HUVEC cells transfected with control siRNA or VEGFR2 siRNA cells cultured in conditioned medium from MCF7 cells stably infected with lentiviruses carrying GATA1 or SET7. Scale bar: 100 µm. (**E**) CAM assays with conditioned medium from MCF7 cells stably infected with lentiviruses carrying GATA1 or SET7 cells and transfected with control siRNA or VEGFR2 siRNA. All values shown are mean ± SD of triplicate measurements and have been repeated 3 times with similar results. *$P < 0.05$, versus corresponding control.

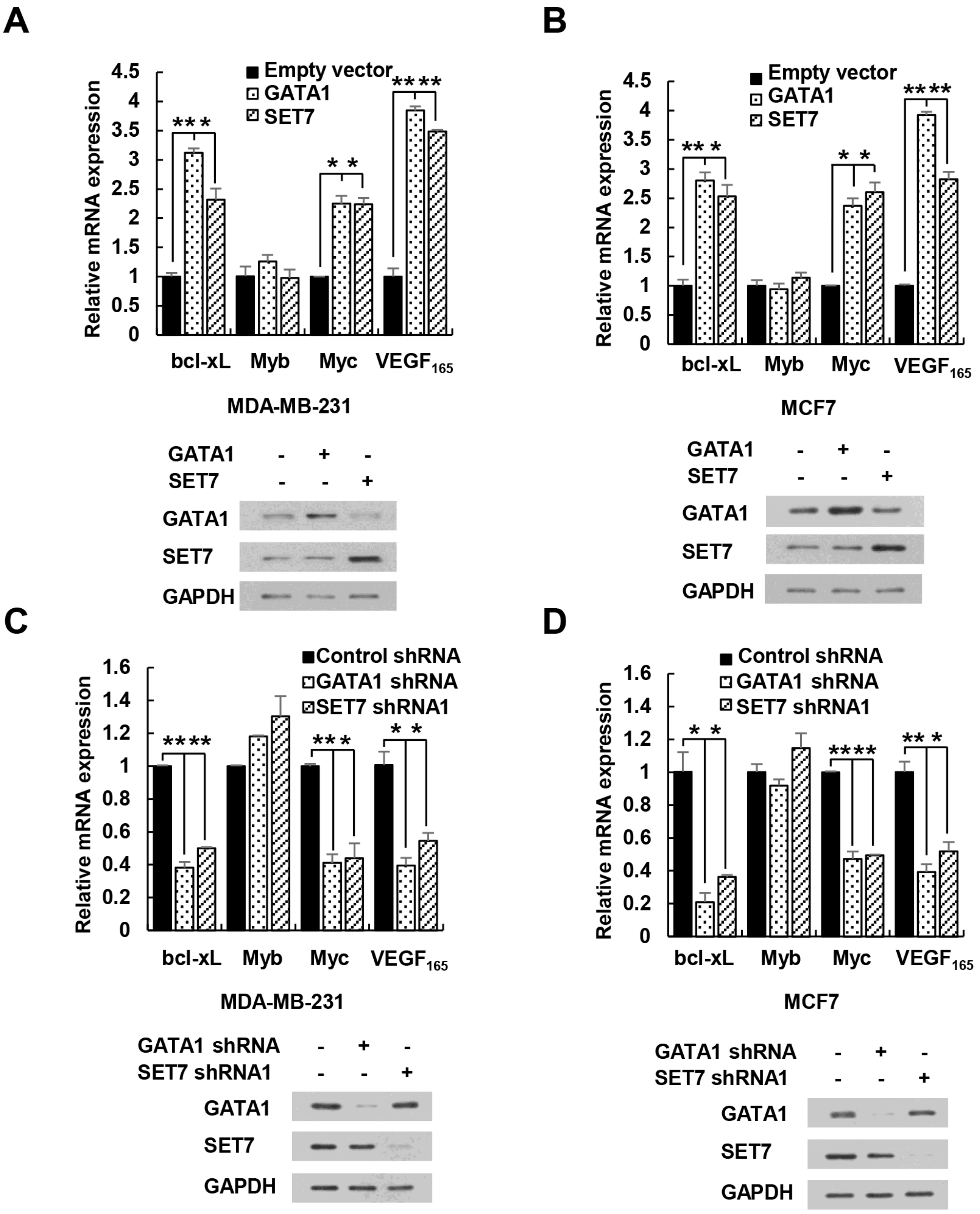

Supplementary Figure S11: GATA1 and SET7 regulate the transcription of known GATA1 target genes. (**A**, **B**) Real-time RT-PCR analysis of the transcription of bcl-xL, Myb and Myc in MDA-MB-231 (A) or MCF7 (B) cells infected with lentiviruses carrying GATA1 or SET7. $VEGF_{165}$ was used as a control for comparison. (**C**, **D**) Real-time RT-PCR analysis of the transcription of bcl-xL, Myb and Myc in MDA-MB-231 (C) or MCF7 (D) cells infected with lentiviruses carrying GATA1 shRNA or SET7 shRNA. Western blot shows the expression of GATA1 and SET7 (A–D). All values shown are mean ± SD of triplicate measurements and have been repeated 3 times with similar results. $^*P < 0.05$, $^{**}P < 0.01$.

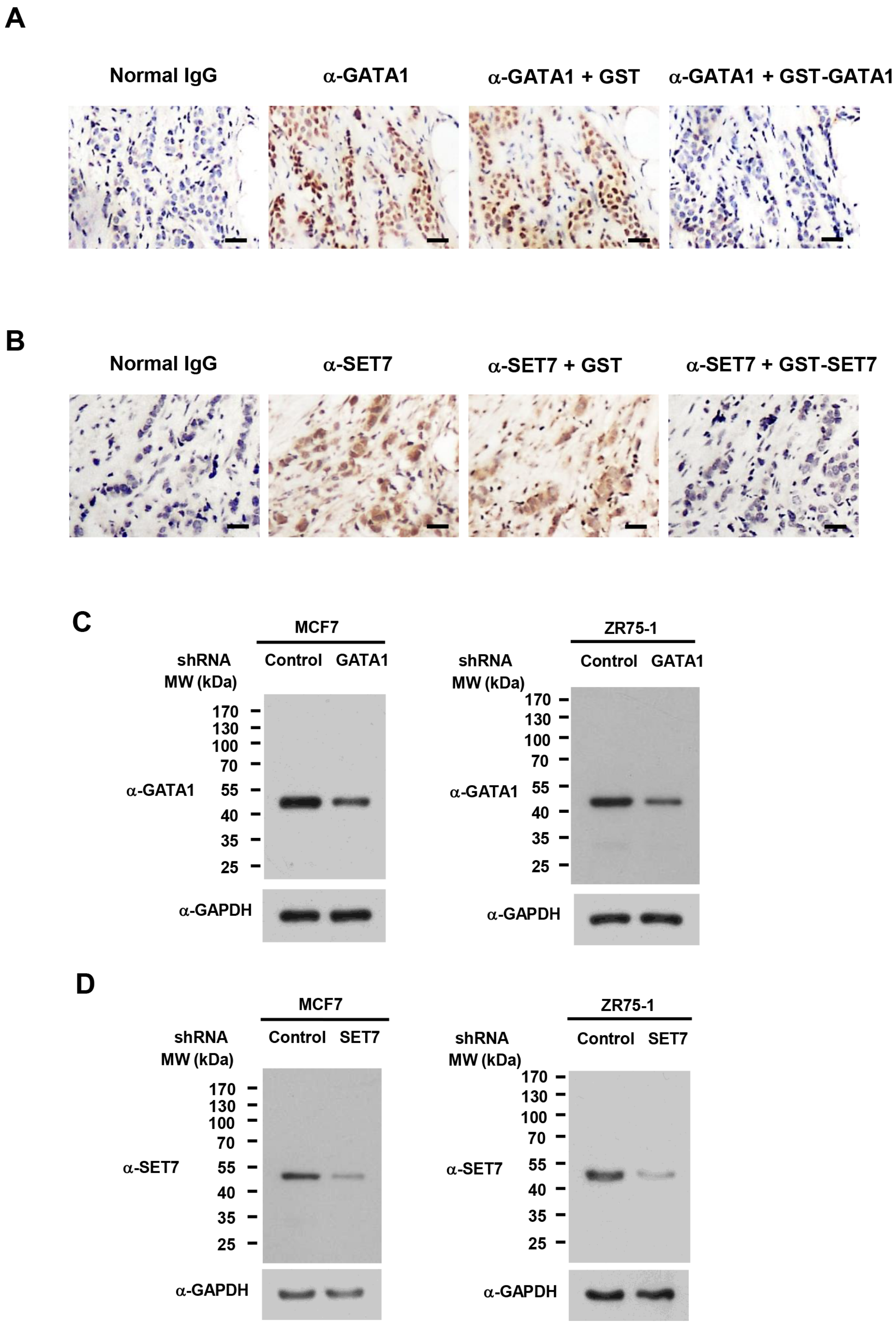

Supplementary Figure S12: Validation of antibody specificity to GATA1 and SET7. (**A**, **B**) Immunohistochemical staining of breast cancer samples incubated with normal IgG, anti-GATA1 (A) or anti-SET7 (B). To validate antibody specificity, the anti-GATA1 or anti-SET7 was pre-incubated with recombinant GST-GATA1 or GST-SET7 protein or GST for 1 h prior to applying to tissue. Scale bar, 25 μm. (**C**, **D**) Western blot analysis of cell lysates from MCF7 (left panel) or ZR75-1 (right panel) cells infected with control shRNA, GATA1 shRNA or SET7 shRNA1 using antibodies specific for anti-GATA1 (C) or anti-SET7 (D).
